# Sub-micrometer focusing setup for high-pressure crystallography at the Extreme Conditions beamline at PETRA III

**DOI:** 10.1107/S1600577522002582

**Published:** 2022-04-04

**Authors:** K. Glazyrin, S. Khandarkhaeva, T. Fedotenko, W. Dong, D. Laniel, F. Seiboth, A. Schropp, J. Garrevoet, D. Brückner, G. Falkenberg, A. Kubec, C. David, M. Wendt, S. Wenz, L. Dubrovinsky, N. Dubrovinskaia, H.-P. Liermann

**Affiliations:** a Deutsches Elektronen-Synchrotron DESY, Notkestrasse 85, 22607 Hamburg, Germany; b Bayerisches Geoinstitut, University of Bayreuth, Universitätsstrasse 30, 95440 Bayreuth, Germany; c Material Physics and Technology at Extreme Conditions, Laboratory of Crystallography, University of Bayreuth, Universitätsstrasse 30, 95440 Bayreuth, Germany; d Center for X-ray and Nano Science CXNS, Deutsches Elektronen-Synchrotron DESY, Notkestrasse 85, 22607 Hamburg, Germany; e Helmholtz Imaging Platform, Deutsches Elektronen-Synchrotron DESY, Notkestrasse 85, 22607 Hamburg, Germany; f Department Physik, Universität Hamburg, Luruper Chaussee 149, 22761 Hamburg, Germany; g Ruhr-Universität Bochum, Universitätsstrasse 150, 44801 Bochum, Germany; h Laboratory for Micro- and Nanotechnology, Paul Scherrer Institut, Forschungsstrasse 111, 5232 Villigen-PSI, Switzerland; i Department of Physics, Chemistry and Biology (IFM), Linköping University, Campus Valla, Fysikhuset F310, SE-581 83 Linköping, Sweden

**Keywords:** high pressure, diamond anvil cells, X-ray diffraction, phase correcting plate, sub-micrometer focusing

## Abstract

Scientific tasks aimed at decoding and characterizing complex systems and processes at high pressures set new challenges for modern X-ray diffraction instrumentation in terms of X-ray flux, focal spot size and sample positioning. Presented here are new developments at the Extreme Conditions beamline (P02.2, PETRA III, DESY, Germany) that enable considerable improvements in data collection at very high pressures and small scattering volumes. In particular, the focusing of the X-ray beam to the sub-micrometer level is described, and control of the aberrations of the focusing compound refractive lenses is made possible with the implementation of a correcting phase plate. This device provides a significant enhancement of the signal-to-noise ratio by conditioning the beam shape profile at the focal spot. A new sample alignment system with a small sphere of confusion enables single-crystal data collection from grains of micrometer to sub-micrometer dimensions subjected to pressures as high as 200 GPa. The combination of the technical development of the optical path and the sample alignment system contributes to research and gives benefits on various levels, including rapid and accurate diffraction mapping of samples with sub-micrometer resolution at multimegabar pressures.

## Abstract 2

Facing the challenges of X-ray diffraction from tiny samples subjected to multimegabar pressures, instrumentation developments are presented that enable, among other studies, single-crystal data collection from micrometer- to sub-micrometer-sized grains. The developments are based on a sub-micrometer beam capability employing compound refractive lenses operating with a phase correcting plate and a precise motorization solution.

## 1. Introduction

The exploration of systems subjected to high-pressure conditions using a combination of various pressurizing devices and a great number of sensing techniques has enabled great progress in the fields of geological and planetary sciences, materials science, solid-state physics and chemistry. In particular, the diamond anvil cell (DAC), where two opposing anvils compress the sample, has been widely used for the characterization of samples in single-crystalline form up to 2 Mbar (1 bar = 100 000 Pa) (Khandarkhaeva *et al.*, 2020 ▸) and as powders at multiple megabars (Dorfman *et al.*, 2012 ▸; Dubrovinskaia *et al.*, 2016 ▸; Dubrovinsky *et al.*, 2015 ▸; Tateno *et al.*, 2010 ▸). More recently, newly developed DAC designs such as toroidal anvils (Dewaele *et al.*, 2018 ▸; Jenei *et al.*, 2018 ▸; McMahon, 2018 ▸) and the double-stage DAC technique (Dubrovinskaia *et al.*, 2016 ▸; Dubrovinsky *et al.*, 2015 ▸, 2012 ▸) have reached pressures up to 0.6 and 1 TPa, respectively. In addition, it has become apparent that the new synthesis products in high-pressure chemistry experiments can only be evaluated efficiently if one can map and isolate the grain of interest from the surroundings, *e.g.* unreacted precursor materials, the pressure medium or other products of the synthesis.

Thus, one of the major challenges of structural studies at conventional and ultra-high pressures is the reduction of sample sizes, which often requires probes with very small beam size and high flux. As X-ray diffraction (XRD) is the most critical and one of the most powerful probes, the demand for sub-micrometer X-ray diffraction capabilities has increased significantly over the last decade. Developments at large-scale facilities are at the forefront of state-of-art studies employing small beams and enable the determination of crystal structures and their evolution under extreme conditions, using either single crystals (single-crystal X-ray diffraction, SCXRD) [*e.g.* Bykov *et al.* (2019 ▸), Bykova *et al.* (2016 ▸), Friedrich *et al.* (2020 ▸), Laniel *et al.* (2020 ▸)] or powders (powder X-ray diffraction, PXRD, Anzellini *et al.*, 2019 ▸; Errandonea *et al.*, 2020 ▸; Kawamura *et al.*, 2002 ▸).

Review of the available literature shows that, in comparison with powder diffraction techniques, high-pressure SCXRD often provides a more reliable pathway for an accurate and precise structure solution, although the process of data acquisition can be more complex. Indeed, sub-micrometer-sized samples, *e.g.* synthesized in a DAC after laser heating, have to stay illuminated by the X-ray beam upon rotation by ±38° or higher angles. At the same time, the signal of interest should not be spoiled by a possible overlap with other phases, or grains of the same phase but different orientations. The centering of micrometer-sized samples requires the implementation of more precise and accurate sample-positioning systems on dedicated high-pressure beamlines. Thus, only a combination of precise motorization with the sub-micrometer beam capability of the instrument enables successful experiments, significantly improving the quality and usability of the data.

Here, we report the development of a sub-micrometer focusing setup on the general-purpose experiment table of beamline P02.2 at PETRA III, DESY, Hamburg, Germany (Liermann *et al.*, 2015 ▸). The new setup has a sub-micrometer focusing achieved by compound refractive lenses (CRLs), supplemented with a phase plate which reduces the spherical aberrations of the CRLs at 25.6 keV. The implementation of a correcting phase plate reduces the beam tails at the focal spot (Seiboth *et al.*, 2017 ▸). This new development significantly improves the capability of the beamline, enabling users to collect data with a beam size at the focal spot of 0.9 µm × 0.9 µm [horizontal (H) × vertical (V) at FWHM] and a photon flux higher than that without the correcting phase plate.

In addition, we describe an improved sample-positioning system with the implementation of a modern air-bearing rotation stage operating in combination with piezo actuator *XY* stages, enabling a spherical confusion of the order of a micrometer or less and a position accuracy of 0.1–0.2 µm. We demonstrate the capability of the new setup as an instrument by performing a test using micrometer-sized single crystals of CoSb_3_ and (Mg_1.93_Fe_0.06_)(Si_1.93_,Al_0.06_)O_6_ (orthoenstatite) prepared by the focused-ion-beam technique (FIB). Our studies, conducted at pressures above 150 GPa, characterize Fe_3_O_4_ and Fe-bearing perovskite Mg_0.91(2)_Fe_0.09(2)_SiO_3_ measured in a multigrain matrix after laser-heating in diamond anvil cells. These state-of-the-art examples highlight the full potential of the setup with application to high-pressure crystallographic studies.

## 2. Implementation of CRLs and a phase plate

A detailed description of the Extreme Conditions Beamline, P02.2, at the third-generation light source PETRA III can be found in the article by Liermann *et al.* (2015 ▸). In order to improve the focusing capabilities of the beamline, *e.g.* 2 µm × 2 µm obtained using a Kirkpatrick–Baez (KB) mirror system, 136 2D parabolic Be CRLs with a radius of curvature at their apex of 50 µm and a geometric aperture of 360 µm were installed at around 70.5 m from the source for X-ray diffraction experiments at 25.6 keV. Using the full X-ray acceptance of the CRLs, a focused beam of 3 µm × 1.2 µm (H × V) was achieved at a focal distance of 395 mm. Here and below, the values are reported for the FWHM. In order to achieve a smaller beam size at the focal spot, we reduced the incident beam to 50 µm × 50 µm at the position of the high heat load slits system (SLT) located in the middle of the beamline about 35 m from the source [just before the double-crystal monochromator (DCM) installed at 40.4 m]. Thus, a small central area of the incident X-ray beam was used for focusing, resulting in a focal spot of ∼0.9 µm × 0.9 µm as determined with sharp-edge scans at the sample position (scans of absorbing cylindrical rods of 6 mm diameter made from hardened steel, in the horizontal and vertical directions in transmission mode).

**Figure 1:**
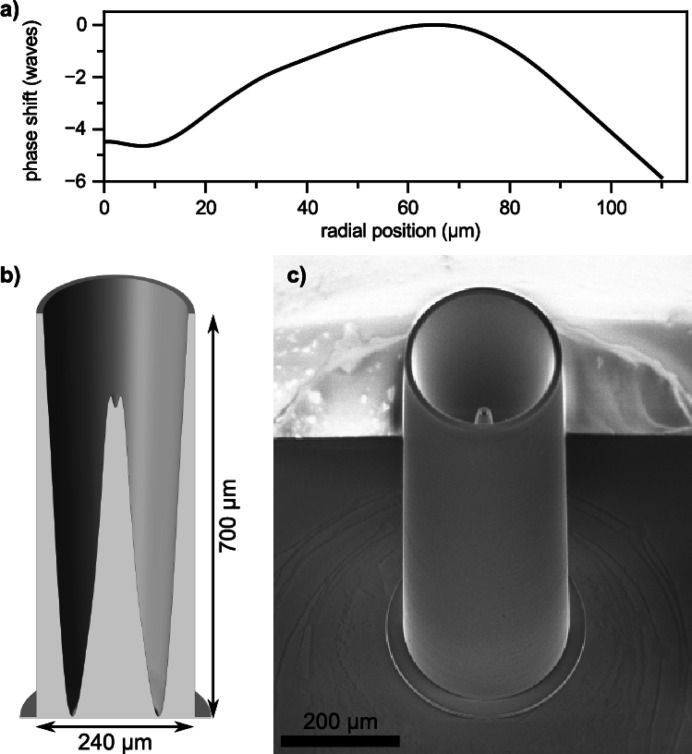
The correcting phase plate and its design. (*a*) The phase shift was quantified for the 136 CRLs on the P06 beamline by means of ptychography. (*b*) The corresponding cross section of a 3D model of the phase plate providing appropriate aberration corrections for this specific CRL stack. (*c*) An SEM image of the phase plate produced on a silicon nitride substrate.

Unfortunately, the size reduction of the incident beam at the position of the SLT also resulted in a significant loss of the focused beam intensity. In order to improve the photon flux at the focal spot, but at the same time keep the beam size to sub-micrometer level, we reduced the spherical aberration of the lenses (Celestre *et al.*, 2020 ▸; Seiboth *et al.*, 2016 ▸) by implementing a customized phase plate downstream from the CRLs. Prior to the phase plate production, a stack of 136 lenses was pre-characterized on the Hard X-ray Micro/Nano-Probe Beamline P06 at PETRA III via ptychography in order to achieve optimal lateral coherence in the horizonal direction at 25.6 keV (Falkenberg *et al.*, 2020 ▸; Seiboth *et al.*, 2020 ▸). The calculated radial phase shift compensating for spherical aberrations is shown in Fig. 1 ▸(*a*). A polymer structure with a height profile that matches the necessary phase correction was fabricated by a two-photon polymerization method. A cross section of the 3D model of the phase plate is shown in Fig. 1 ▸(*b*). The structure was created on a silicon nitride membrane with a Nanoscribe Photonic Professional GT using a ‘dip-in’ lithography mode with a 25× objective (numerical aperture NA = 0.8). Fig. 1 ▸(*c*) shows a scanning electron microscopy (SEM) image of the phase plate tailored to reduce the characteristic spherical aberration of the Be CRL stack used on P02.2.

**Figure 2:**
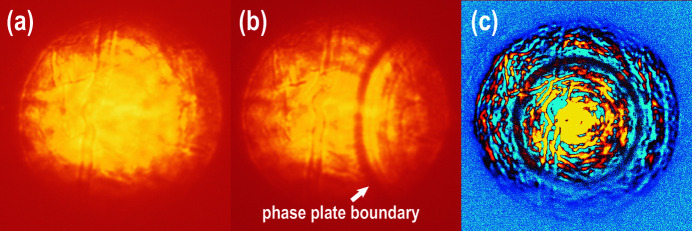
The phase plate alignment procedure. The phase plate is located between the CRLs and the microscope installed downstream from the focal spot position. (*a*) An image of a defocused X-ray beam without the phase plate. (*b*) The phase plate is moving in the field of view, approaching the central position from the right-hand side. (*c*) The phase plate is centered and the image is produced by subtracting the background, *e.g.* panel (*a*), from the image with the phase plate centered. In the final steps of alignment and with the sharp edge placed at the position of optimal focus, we make a small displacement of the phase plate perpendicular to the incident beam and scan the sharp edge in order to achieve the best beam shape.

Considering the setup of P02.2, the phase plate was placed 29 mm downstream from the edge of the lens stack installed in the housing of a V-grooved CRL holder (Lengeler *et al.*, 2005 ▸). The CRL holder provides an inert He environment protecting the CRLs from oxidation. The phase plate was installed externally. To align the custom-made correcting phase plate to the optical axis of the CRL stack in the horizontal and vertical directions, a motorized positioning system consisting of 2MFA-PP micro-steppers from Newport was mounted on the CRL V-groove housing. A high-resolution scintillator-based Optique Peter microscope (PCO.edge 4.2 CLHS camera, 20× objective, 10 µm thick YAG:Ce scintillator) was used to observe and control the alignment of the phase plate (Fig. 2 ▸).

The lenses of the CRL stack were numbered during our measurement at P06 and were installed on the beamline in the same order. This step is important, since the phase shift of the correcting phase plate was calculated for a specific lens configuration. After alignment of the CRLs and the correcting phase plate, further optimization steps were carried out in search of a compromise between the photon flux and the beam size at the focal spot. This included iterative adjustments of the SLT opening and the tracking of the resulting beam size at the optimal focal spot. We measured the latter by means of sharp-edge scans at different focal distances. In the end, the incident beam size at the SLT had dimensions of ∼150 µm × 150 µm and the corresponding optimal focal distance was 400–402 mm (the center of the CRL stack to the focal point).

**Figure 3:**
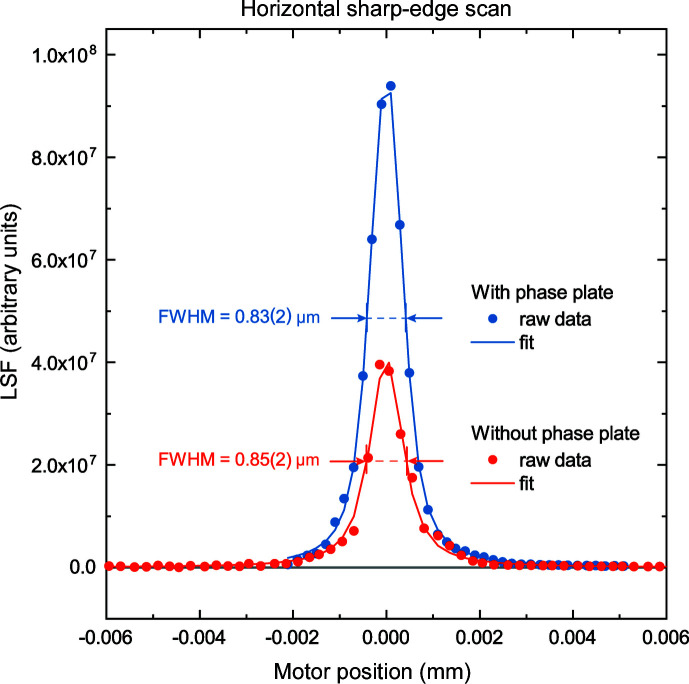
A comparison of the horizontal focal size with (blue data) and without (red data) the correcting phase plate. The curves correspond to the derivative signal of the sharp-edge scans, the linear spread function (LSF). Analysis of the curves confirms the similar size of the X-ray beam in the horizontal direction. Given the same acquisition time per point, the higher values for the blue data indicate higher flux values for the setup when corrected with the phase plate. With the phase plate (blue), one can employ a larger opening of the SLT installed in front of the DCM position. Implementation of the phase plate enables a higher intensity X-ray beam while maintaining a small beam size with similar, or even slightly smaller, beam tails at the focal spot.

By scanning the X-ray beam at the focal spot using an absorbing edge we measured a transmission curve with a sigmoid-like shape which corresponds to an edge spread function (ESF) (Wang *et al.*, 2016 ▸, and references within). By derivation of the ESF we produced a linear spread function (LSF) response enabling determination of the focal spot size. In order to assess the situation before and after the installation of the phase plate, and to take advantage of using the latter, we collected data which are presented in Fig. 3 ▸. For case (i), the red line depicts the beam shape at the focal spot without the phase plate and with a smaller opening of the SLT (50 µm × 50 µm), while for case (ii) the blue line shows the data with the phase plate installed and a larger opening of the SLT (∼150 µm × 150 µm). Considering case (i), we add that further SLT opening without installation of the phase plate increases the focal spot beyond 1 µm. Raw data for the curves were measured using the same pin-diode acquisition time, so the installation of the phase plate has produced a gain with a factor of ∼2.2. Comparing cases (i) and (ii), we conclude that a similar focal spot size in the horizontal direction was achieved in both cases, including similar or lower beam tail contributions, but installation of the phase plate enabled a higher photon flux.

**Figure 4:**
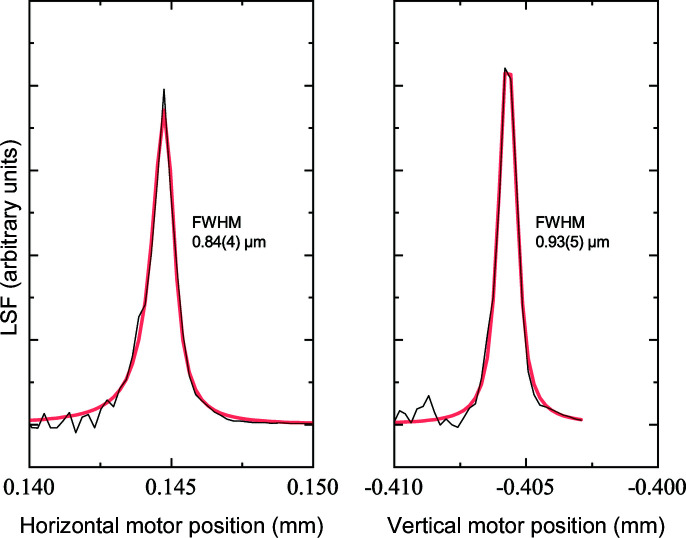
First-order derivatives of sharp-edge scans (LSF) collected during (left) horizontal and (right) vertical motor movement. The FWHM is shown together with the corresponding error bar estimates.

In one of the final steps, the sample stack position was readjusted in order to bring the position of the sample stack rotation axis (controlling the sample-to-detector distance, SDD) into focus. Fig. 4 ▸ illustrates the horizontal and vertical beam profiles in the focal place used for the measurement of the single-crystal data reported below. The optimization of the X-ray optical path included the installation of a Pt-based pinhole, ranging from 15 to 40 µm in diameter depending on the application. The photon flux for the ‘sub-micrometer’ setup at the beam position with a 40 µm pinhole was measured to be around 3.7 × 10^9^ photons s^−1^ using a calibrated passivated implanted planar silicon (PIPS) diode.

The opening of the SLT does have several effects on the X-ray optical path. On one hand, it controls the total energy dissipating at the DCM crystals (*e.g.* heat bump, non-ideal surface of crystals). On the other hand, the closing of the SLT effectively introduces a secondary source. It is hard to give a quantitative description of the realistic and non-ideal system, an analysis of which goes beyond the current publication. However, we note that, after further opening of the SLT, an increase in flux by at least of a factor 3–5 was observed. At the same time, the beam size at the focal spot increased to 1.3 µm × 1 µm (H × V) FWHM. This option may be of interest to user groups working with low-*Z* materials, where the compromise between the illuminating beam intensity and the beam size at the focal spot is shifted towards the higher photon flux at the sample position.

## 3. Sample positioning system with sub-micrometer resolution

**Figure 5:**
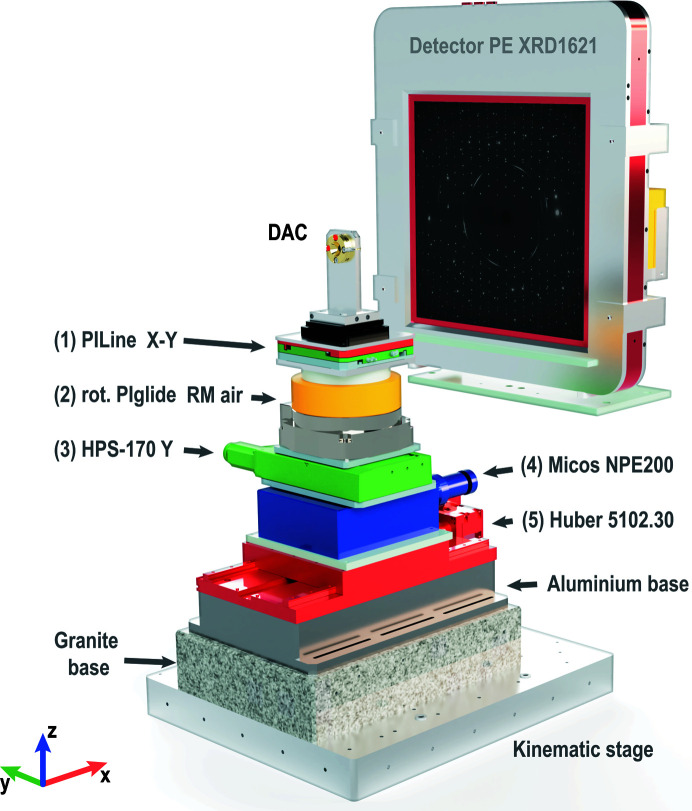
A schematic illustration of the sub-micrometer motor sample stack. ‘DAC’ indicates the position of a sample mounted in a DAC. For clarity, individual components are represented by different colors and are numbered. The corresponding coordinate system employed at the beamline is shown at the bottom of the figure. The *x* axis is collinear to the X-ray beam direction. The Perkin–Elmer XRD1621 detector is shown for reference.

Accurate sample positioning is a crucial aspect of a high-pressure DAC experimental setup. Along with the improved focusing, it was necessary to update the existing sample positioning system (Liermann *et al.*, 2015 ▸). We indicate the new parts in Fig. 5 ▸, labelled (1) to (5). An *XY* PILine piezo positioning stage (1) and a PIglide RM air-bearing rotation stage (2) were acquired from Physik Instrumente (PI) GmbH & Co. They replace the top portion of the standard sample six-motor positioning system (Liermann *et al.*, 2015 ▸). In addition, we exchanged the motor (3) that controls the position of the rotation axis and moves it in the horizontal plane perpendicular to the X-ray beam. It was upgraded through an HPS-170 high-precision linear stage also manufactured by PI. The factory characteristics of these positioning units are listed in Table 1 ▸.

As indicated in Fig. 5 ▸, the sample stack is set up on the kinematic mounting plate. It has three cavities matching the three ball point joints mounted on the base granite installed on the beamline. After the installation, the kinematic plate is fixed to the underlying granite by means of several M12 screws. The change from a standard setup (also based on the kinematic stage approach) to the sub-micrometer configuration takes a few hours, including motor reconnection and reconfiguration. Optimal operation parameters of the motors were determined during the commissioning, *e.g.* the *XY* cross-roller stage was optimized for 0.2 nm reproducibility of movement. Loading capacities given in Table 1 ▸ indicate that the sub-micrometer sample stack is not compatible with heavy DAC environments, *e.g.* a vacuum chamber, which is typical for the standard setup. However, the sub-micrometer sample stack can easily hold DACs of various popular designs (*e.g.* symmetric, BX90, Boehler–Almax *etc.*), including a membrane connected to a pressure controller. The positioning of the samples by means of the sub-micrometer motor stack is fully integrated into the beamline software. It is fully compatible with our standard setup (*e.g.* synchronization of sample positions by means of offline and online microscopes available for users).

Below we discuss the performance of the sub-micrometer instrument setup from a user’s perspective, with several examples of single-crystal studies under ambient and high-pressure conditions.

## 4. Performance of the sub-micrometer experimental setup with samples under ambient conditions

Two single crystals with different absorption lengths (high and low *Z*) were used to test the accuracy and precision of the sample-positioning system: orthoenstatite [(Mg_1.93_,Fe_0.06_)(Si_1.93_,Al_0.06_)O_6_, *Pbca*, *a* = 18.2391 (3), *b* = 8.8117 (2), *c* = 5.18320 (10) Å] and CoSb_3_ [ 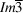 , *a* = 9.0357 (3) Å], measured at Bayerisches Geoinstitute using the eight-position centering procedure (Angel & Finger, 2011 ▸). Both materials are stable under ambient conditions and have large unit cells that produce a considerable number of reflections, which is ideal for testing.

**Figure 6:**
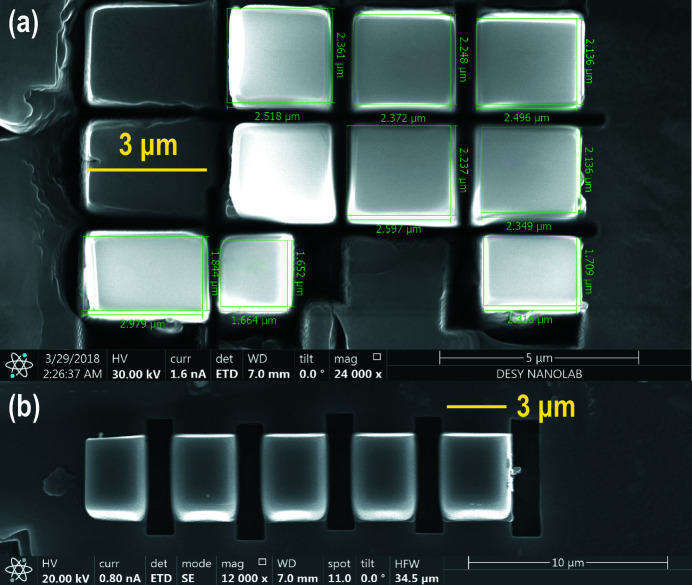
SEM images produced by the SCIOS dual-beam FIB at the NanoLab. (*a*) Prepared pieces of orthoenstatite. (*b*) CoSb_3_ single crystals.

Tiny single crystals of orthoenstantite and CoSb_3_ were prepared using an FIB instrument (FEI SCIOS dual beam) located at DESY NanoLab. The pre-cut single-crystalline lamellae were reduced to smaller sizes with lateral dimensions of 2–4 µm (Fig. 6 ▸). These grains were transferred by means of a Microsupport Axis Pro micromanipulator into DACs used as sample holders, ensuring sample safety in transit. We employed a Re gasket of ∼10 µm thickness indented by 40 µm culet diamond anvils of Boehler–Almax design. These test crystals were measured under ambient conditions.

Single-crystal samples were measured using a Perkin–Elmer XRD1621 amorphous silicon detector bonded to a CsI scintillator. During the data acquisition, the crystals were rotated by ±32° and ±38° with a step size of 0.5° for CoSb_3_ and orthoenstatite, respectively. The wavelength of the X-ray beam was tuned to 0.4830 Å (25.67 keV). Alignment of the sample to the center of the sub-micrometer motor stack rotation was performed with the help of X-ray transmission scans. We used the CoSb_3_ absorption signal as a reference, while in the case of orthoenstatite (separate DAC, low-*Z* material) we had to use the absorption signal from a thin gasket and then locate the orthoenstatite crystal using a visible light microscope on the beamline.

**Figure 7:**
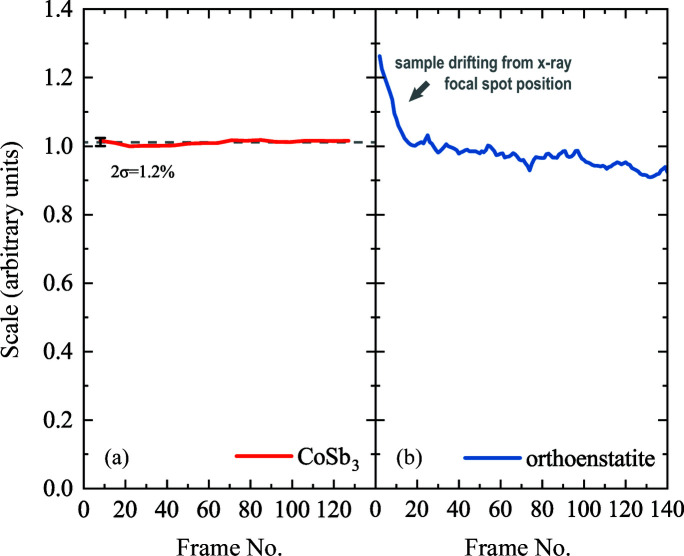
The output of the SCALE3 ABSPACK empirical absorption correction routine of *CrysAlis PRO*, indicating the amount of scaling which had to be applied to each frame in order to compensate for the intensity mismatch between different Friedel equivalents. Each frame corresponds to a physical angle with a step size of 0.5° per frame. (*a*) The flat red line with 2σ = 1.2% is attributed to an average value of 1.011 (gray dashed line). It indicates the excellent stability of the CoSb_3_ sample with respect to the X-ray beam during single-crystal data acquisition. (*b*) Our analysis shows that the orthoenstatite single crystal was moving slightly out of the X-ray beam in the angular range corresponding to frame Nos. 0–20. We consider that the centering procedure involving the gasket absorption profile combined with visible light observations was not perfect enough. The difference in point scatter between the red and blue lines can be attributed to the difference in scattering factors. For the data in panel (*a*) we had to use a 50 µm Pt absorber foil in order to reduce the intensity of the diffraction signal coming from the sample. The much stronger scattering from CoSb_3_ than from orthoenstatite is the reason for the noise difference between the red and blue curves.

Data were processed using the *CrysAlis PRO* software package (Rigaku, 2019 ▸), including the SCALE3 ABSPACK routine for empirical absorption correction and conventional single-crystal data treatment. The output of the program demonstrates the exceptional sample stability during the data collection of CoSb_3_ (Fig. 7 ▸). A minor deviation was detected in the case of the orthoenstatite sample, and we attribute this to the difficulty of sample centering for this poor X-ray scattering material.

**Figure 8:**
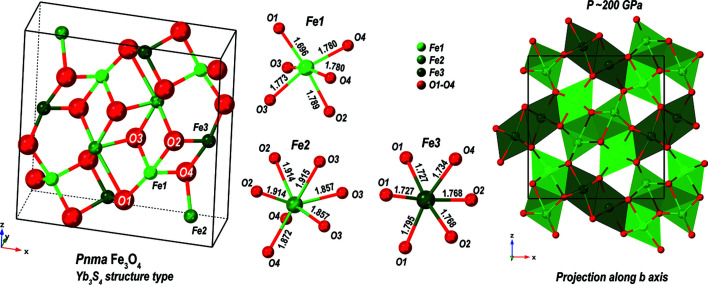
The crystal structure of *Pnma* Fe_3_O_4_ formed after laser heating at 3000–3500 K. The structure is composed of layers of distorted octahedra (Fe1 and Fe3 sites) which are interconnected through the capped trigonal prisms (Fe2 sites). The structure solution converged to *R*(*F* ^2^) = 0.071, *wR*(*F* ^2^) = 0.075 using reflections with an intensity *I* > 2σ(*I*). The individual building blocks and the corresponding Fe—O bonds are shown. On the right-hand side is a projection of the structure along the *b* axis, providing a clear indication of edge sharing of the corresponding building blocks and an overview of the Fe–O framework.

Structure solution and refinement were conducted using *OLEX2* (Dolomanov *et al.*, 2009 ▸) with the *SHELX* backend (Sheldrick, 2008 ▸, 2015 ▸) and the *JANA2006* package (Petříček *et al.*, 2006 ▸). The refinement parameters and the corresponding structural information are summarized in Table 2 ▸. Additional information, including CIFs, is provided in the supporting information. Illustrations of the crystal structure in Fig. 8 ▸ were prepared with the *CrystalMaker* software (Palmer, 2014 ▸).

The data quality reported in Table 2 ▸ is exceptional and in very good agreement with the literature data, even though the energy of the X-ray beam was very low (25.67 keV) which significantly limits our access to reciprocal space due to the finite aperture of DACs. Future developments and enhancements expected for diffraction-limited storage rings (DLSRs), such as the ESRF EBS, APS-U and PETRA IV, will boost these types of studies. Considering the ECB at PETRA III, the upgrade to PETRA IV (Schroer *et al.*, 2019 ▸) promises improved capabilities. The upgrade will result in photon flux improvement at all photon energies interesting for high-pressure research and, in combination with sub-micrometer focusing, it will exceed the capabilities of third-generation sources.

## 5. Applications to materials compressed above ∼150 GPa in laser-heated DACs

A successful crystallographic solution for the test cases described below would not be possible with our standard motor setup. The implementation of the new motorization enabled more accurate and reproducible sample positioning in 3D space, including sample positioning at the center of rotation of the highly accurate rotation stage. Due to the improvements in the motorization and the implementation of the correcting phase plate, which reduces the overload of the sample signal in reciprocal space, the resulting data quality was greatly improved. Each of the described cases represents an important challenge for various fields of science, such as crystal chemistry, mineral physics and planetary sciences.

Iron oxides are closely related to planetary evolution (Bykova *et al.*, 2016 ▸; Dobson & Brodholt, 2005 ▸), but also play a significant role in thermal energy storage, as indicated by various studies (Grosu *et al.*, 2017 ▸; Huang & Xu, 2019 ▸). High-pressure high-temperature treatment of the Fe–O system at elevated pressures induces various phases and structural transformations. Below we report on the observation of the *Pnma* phase of Fe_3_O_4_ at ∼200 GPa. The starting material, α-Fe_2_O_3_ (hematite), was loaded into the sample chamber of a BX90 DAC (Kantor *et al.*, 2012 ▸) together with Ne as the pressure-transmitting medium (Kurnosov *et al.*, 2008 ▸). The starting material was laser-heated at the Bayerisches Geo­institut using a laser-heating system recently developed by Fedotenko *et al.* (2019 ▸). This is a pulsed laser-heating system operating in continuous mode (NIR laser beam, 1070 nm, 5 µm at FWHM in diameter). Pressure was measured using the equation of state of Ne (Fei *et al.*, 2007 ▸). Single-crystal datasets were collected using the Perkin–Elmer XRD1621 detector with a wavelength of 0.483 Å. A complete description of the structure of *Pnma* Fe_3_O_4_ observed at 200 GPa, including a CIF, is provided in the supporting information. The small X-ray beam size was crucial as it constrained the number of illuminated grains and the overlay of scattered intensity in reciprocal space originating from various sources in the high-pressure environment.

The laser heating of the compressed hematite produced a polycrystalline mixture. Analysis of 2D scanning map data of the laser-heated area indicated several larger single-crystal grains that are potential candidates for structural solution. The indexing of the reflections belonging to two strongly scattering *Pnma* Fe_3_O_4_ (oP-Fe_3_O_4_) domains was identified after careful inspection of reciprocal space using the *CrysAlis PRO* software. A more detailed description of the process is provided in the supporting information. The lateral size of the domains within the 2D map did not exceed a couple of micrometers. The integration of intensities for the most strongly scattering domain converged with *R*_int_ = 0.029. The structure was solved and refined based on 195 measured reflections for 22 refined parameters with *R*_1_ = 0.071 (Table 3 ▸). The structure of oP-Fe_3_O_4_ contains two different octahedrally coordinated positions of iron atoms (Fe1 and Fe3) with the Fe—O bond distance varying between ∼1.70 and 1.80 Å (Fig. 8 ▸). The distorted octahedra share edges and form ribbons, and two enantiomorphic ribbons are joined together, hinging along lines of the shared oxygen parallel to the *c* axis. The Fe2 atom is surrounded by seven oxygen atoms with an Fe—O distance variation of ∼1.86–1.91 Å. Its coordination can be considered as a capped trigonal prism. The structure of novel oP-Fe_3_O_4_ belongs to the Yb_3_S_4_ structure type (Chevallier *et al.*, 1967 ▸). It is common for warwickites, mixed metal borates of the general formula *A*_*x*_ *T*_4–*x*_B_2_O_8_, where *A* and *T* are alkaline earths, lanthanides or transition metals (Matos *et al.*, 1996 ▸).

Charge-balance considerations suggest that the Fe1 and Fe3 sites are occupied by Fe^3+^. Our analysis of the Fe1—O and Fe3—O distances, supplemented by analysis of the octahedron site volumes, reveals that the Fe1 and Fe3 atoms are in a low-spin state (Vasiukov, 2018 ▸). The Fe2 site is attributed to Fe^2+^. It is clearly larger than the Fe1 and Fe3 sites. We suggest that the spin configuration of Fe2 should be also attributed to low spin. However, due to the deformation of the crystallographic site we cannot fully exclude the possibility of an intermediate spin-state configuration. The precise characterization of the iron atoms’ electronic state in oP-Fe_3_O_4_ and the exploration of its phase diagram require further investigation.

The Mg–Si–O system is equally if not more important than Fe–O for our understanding of the Earth and other planetary environments. In addition to the example of the Fe_3_O_4_ system, we show a full structure solution and refinement of excellent quality for Fe-bearing bridgmanite Mg_0.91 (2)_Fe_0.09 (2)_SiO_3_. The data were obtained after laser heating and collected at a pressure of ∼155 GPa (Ne pressure scale; Fei *et al.*, 2007 ▸). Accurate measurements of the physical properties of bridgmanite and post-perovskite, including density as a function of pressure, temperature and composition, have always been a focus of solid-earth investigations. The studies continue with a doubled effort today involving static and dynamic compression methods [*e.g.* Fei *et al.* (2021 ▸)]. However, information characterizing the density alone can rightfully be considered insufficient. It must be supplemented with a thorough understanding of the crystal structure and its evolution and perturbation. Here, ultra-high-pressure studies of the Mg–Si–O system by means of SCXRD represent a very specific but very important challenge. Most experimental difficulties could be tracked down to the low scattering volumes of the sub-micrometer-sized samples, but they become even more severe due to the low atomic scattering factor in the case of the Mg–Si–O system. Using the example of bridgmanite laser-heated to 2500–3000 K, we have demonstrated the power of the new sub-micrometer setup on P02.2, enabling structural studies of chemically complex systems at ultra-high pressures, even those containing mostly light elements in their composition (*e.g.* Mg, Si, O). As we show in Table 3 ▸, the final refinement of the structural model converges with a very low *R* factor of *R*_1_ = 0.0411 based on 106 measured reflections and 13 refined parameters.

## 6. Conclusions

By applying the most recent technical developments within the capabilities of PETRA III, we report an upgrade of the existing high-pressure micro-X-ray diffraction instrument at P02.2. The upgrade involves the modification of the X-ray optical path and the implementation of a phase plate correcting the spherical aberration of the CRLs implemented at P02.2 in combination with the new, more accurate and precise, motorization of the sample stack.

Within our test studies conducted under ambient conditions and at pressures exceeding 1 Mbar, we have demonstrated the most challenging issues faced in high-pressure science and presented a solution addressing the challenges of single-crystal refinement from grains of micrometer to sub-micrometer dimensions. We have described ‘synthetic’ examples of orthoenstatite and CoSb_3_ measured inside the DAC under ambient conditions and extended our studies to scientific cases exploring the crystal structures of Fe_3_O_4_ and Mg_0.91 (2)_Fe_0.09 (2)_SiO_3_. The structures of the latter two materials were successfully solved at pressures exceeding 1 Mbar, even though the data acquisition and analysis processes were highly challenging, *e.g.* complicated by sample finding and by signal extraction from micrometer-sized polycrystalline aggregates. The real case studies of Fe_3_O_4_ and Mg_0.91 (2)_Fe_0.09 (2)_SiO_3_ demonstrate the capacity of the current instrument upgrade.

We hope that our study will inspire the high-pressure community and engender further developments on dedicated high-pressure beamlines, extending the capabilities of the latter and driving high-pressure science beyond the boundaries of the commonly explored *P*–*T* space.

## 7. Related literature

For further literature related to the supporting information, see Bykova (2015 ▸), Hrubiak *et al.* (2019 ▸), Petříček *et al.* (2014 ▸), Prescher & Prakapenka (2015 ▸) and Rigaku Oxford Diffraction (2020 ▸).

## Supplementary Material

Crystal structure: contains datablock(s) Mg1.94Fe0.067Si1.93Al0.067O6, I. DOI: 10.1107/S1600577522002582/ve5153sup1.cif

Structure factors: contains datablock(s) I. DOI: 10.1107/S1600577522002582/ve5153Isup2.hkl

Description of the process of single-crystal data collection with an example of oP-Fe3O4. DOI: 10.1107/S1600577522002582/ve5153sup3.pdf

Zip archive of CIF files and FCF files representing full data solutions from materials described in the manuscript, sorted according to their appearance in the text: (i) Enstatite single crystal, ambient conditions, Table 2, (ii) CoSb3 single crystal, ambient conditions, Table 2, (iii) oP-Fe3O4 - ~200 GPa, Table 3, (iv) bridgmanite- ~155 GPa, Table 2. The CIF files were tested using the CheckCIF service of IUCr and the reports are included. DOI: 10.1107/S1600577522002582/ve5153sup4.zip

CCDC reference: 2156936

## Figures and Tables

**Table 1:** Factory characteristics of the new positioning parts used for the sub-micrometer setup on the Extreme Conditions Beamline (ECB) The numbers in brackets refer to components in Fig. 5 ▸.

(1) *XY* stage with PILine piezo motors	25 mm × 25 mm travel range
	100 nm min. incremental motion
	10 nm sensor resolution
	7 N drive force, maximum load capacity 50 N

(2) PIglide RM air-bearing rotation stage	150 mm motion platform diameter
	50 mm travel length
	0.0015 µrad sensor resolution
	Absolute angle-measuring system
	Slot-less brushless three-phase torque motor

(3) HPS-170 high-precision linear stage	170 mm width, 52 mm travel range
	50 nm minimum incremental motion
	Stepper motor
	Linear encoder with sin/cos signal transmission
	Optical limit switches
	350 N load capacity

**Table 2:** Details of the crystal structure refinements of the test samples orthoenstatite (Mg_1.93_,Fe_0.06_)(Si_1.93_,Al_0.06_)O_6_ and CoSb_3_ Atomic displacement parameters of the cations were refined with anisotropic approximation. For additional information on the crystal structures and refinement parameters we refer the reader to the CIFs in the supporting information.

Crystallographic data		
Chemical formula	(Mg_1.94_Fe_0.067_)(Si_1.93_Al_0.067_)O_6_	CoSb_3_
*M*_r_	202.9	424.2
Crystal system, space group	Orthorhombic, *Pbca*	Cubic, *Im*3
Temperature (K)	293	293
*a*, *b*, *c* (Å)	5.1815 (2), 18.2321 (11), 8.8085 (5)	9.0360 (1)
*V* (Å^3^)	832.14 (8)	737.78 (1)
*Z*	8	8
Radiation type	Synchrotron, λ = 0.483 Å	Synchrotron, λ = 0.483 Å
μ (mm^−1^)	0.44	8.83
Crystal size (µm^3^)	4 × 4 × 2	3 × 3 × 2
		
Data collection		
Diffractometer	Single-circle (ω) diffractometer	
Absorption correction	Multi-scan, *CrysAlis PRO* 1.171.40.67a (Rigaku, 2019 ▸), empirical absorption correction using spherical harmonics, implemented in the SCALE3 ABSPACK scaling algorithm	
*T*_min_, *T*_max_	0.824, 1	0.888, 1
No. of measured, independent and observed [*I* > 2σ(*I*)] reflections	1072, 454, 409	490, 133, 121
*R*_int_	0.017	0.016
(sin θ/λ)_max_ (Å^−1^)	0.652	0.645
		
Refinement		
*R*[*F* ^2^ > 2σ(*F* ^2^)], *wR*(*F* ^2^)	0.025, 0.039	0.012, 0.043
No. of reflections	454	133
No. of parameters	61	9
Δρ_max_, Δρ_min_ (e Å^−3^)	0.58, −0.62	1.28, −1.03

**Table 3:** Detailed information for the crystal structure refinements of oP-Fe_3_O_4_ and Mg_0.91 (2)_Fe_0.09 (2)_SiO_3_ performed on laser-heated samples at ultra-high pressures Samples were measured at ambient temperature after a laser-heating procedure. For additional information on the crystal structures and refinement parameters we refer the reader to the CIFs in the supporting information.

Crystal data		
Chemical formula	Fe_3_O_4_	Mg_0.91 (2)_Fe_0.09 (2)_SiO_3_
Pressure (GPa)	∼200	∼155
*M*_r_	231.5	103.3
Crystal system, space group	Orthorhombic, *Pnma*	Orthorhombic, *Pbnm*
Temperature (K)	293	293
*a*, *b*, *c* (Å)	7.932 (10), 2.5881 (13), 8.321 (3)	4.194 (2), 4.525 (1), 6.1910 (1)
*V* (Å^3^)	170.8 (2)	117.49 (6)
*Z*	4	4
Radiation type	Synchrotron, λ = 0.483 Å	Synchrotron, λ = 0.483 Å
μ (mm^−1^)	7.97	0.98
Crystal size (µm^3^)	1 × 2 × 1†	2 × 2 × 2†
		
Data collection		
Diffractometer	Single-circle (ω) diffractometer	
Absorption correction	Multi-scan, *CrysAlis PRO* 1.171.40.67a (Rigaku, 2019 ▸), empirical absorption correction using spherical harmonics, implemented in the SCALE3 ABSPACK scaling algorithm	
*T*_min_, *T*_max_	0.559, 1	0.664, 1
No. of measured, independent and observed [*I* > 2σ(*I*)] reflections	195, 105, 88	106, 56, 53
*R*_int_	0.029	0.008
(sin θ/λ)_max_ (Å^−1^)	0.643	0.607
		
Refinement		
*R*[*F* ^2^ > 2σ(*F* ^2^)], *wR*(*F* ^2^)	0.071, 0.075	0.041, 0.114
No. of reflections	105	56
No. of parameters	22	13
Δρ_max_, Δρ_min_ (e Å^−3^)	2.04, −1.8	0.51, −0.54

† Approximate sizes based on X-ray diffraction 2D mapping.

## References

[bb1] Angel, R. J. & Finger, L. W. (2011). *J. Appl. Cryst.* **44**, 247–251.

[bb2] Anzellini, S., Monteseguro, V., Bandiello, E., Dewaele, A., Burakovsky, L. & Errandonea, D. (2019). *Sci. Rep.* **9**, 13034. 10.1038/s41598-019-49676-y PMC6736956 31506567

[bb45] Bykova, E. (2015). PhD thesis. University of Bayreuth, Germany. https://epub.uni-bayreuth.de/2124/

[bb3] Bykova, E., Dubrovinsky, L., Dubrovinskaia, N., Bykov, M., McCammon, C., Ovsyannikov, S. V., Liermann, H., Kupenko, I., Chumakov, A. I., Rüffer, R., Hanfland, M. & Prakapenka, V. (2016). *Nat. Commun.* **7**, 10661. 10.1038/ncomms10661 PMC4753252 26864300

[bb4] Bykov, M., Chariton, S., Fei, H., Fedotenko, T., Aprilis, G., Ponomareva, A. V., Tasnádi, F., Abrikosov, I. A., Merle, B., Feldner, P., Vogel, S., Schnick, W., Prakapenka, V. B., Greenberg, E., Hanfland, M., Pakhomova, A., Liermann, H., Katsura, T., Dubrovinskaia, N. & Dubrovinsky, L. (2019). *Nat. Commun.* **10**, 2994. 10.1038/s41467-019-10995-3 PMC6611777 31278267

[bb5] Celestre, R., Berujon, S., Roth, T., Sanchez del Rio, M. & Barrett, R. (2020). *J. Synchrotron Rad.* **27**, 305–318. 10.1107/S1600577519017235 PMC7842213 32153269

[bb6] Chevallier, R., Laruelle, P. & Flahaut, J. (1967). *Bull. Soc. Fr. Mineral. Cristallogr.* **90**, 564–574.

[bb7] Dewaele, A., Loubeyre, P., Occelli, F., Marie, O. & Mezouar, M. (2018). *Nat. Commun.* **9**, 2913. 10.1038/s41467-018-05294-2 PMC6060175 30046093

[bb8] Dobson, D. P. & Brodholt, J. P. (2005). *Nature*, **434**, 371–374. 10.1038/nature03430 15772658

[bb9] Dolomanov, O. V., Bourhis, L. J., Gildea, R. J., Howard, J. A. K. & Puschmann, H. (2009). *J. Appl. Cryst.* **42**, 339–341.

[bb10] Dorfman, S. M., Prakapenka, V. B., Meng, Y. & Duffy, T. S. (2012). *J. Geophys. Res.* **117**, B08210.

[bb11] Dubrovinskaia, N., Dubrovinsky, L., Solopova, N. A., Abakumov, A., Turner, S., Hanfland, M., Bykova, E., Bykov, M., Prescher, C., Prakapenka, V. B., Petitgirard, S., Chuvashova, I., Gasharova, B., Mathis, Y., Ershov, P., Snigireva, I. & Snigirev, A. (2016). *Sci. Adv.* **2**, e1600341. 10.1126/sciadv.1600341 PMC4956398 27453944

[bb13] Dubrovinsky, L., Dubrovinskaia, N., Bykova, E., Bykov, M., Prakapenka, V., Prescher, C., Glazyrin, K., Liermann, H.-P., Hanfland, M., Ekholm, M., Feng, Q., Pourovskii, L. V., Katsnelson, M. I., Wills, J. M. & Abrikosov, I. A. (2015). *Nature*, **525**, 226–229. 10.1038/nature14681 26302297

[bb12] Dubrovinsky, L., Dubrovinskaia, N., Prakapenka, V. B. & Abakumov, A. M. (2012). *Nat. Commun.* **3**, 1163. 10.1038/ncomms2160 PMC3493652 23093199

[bb14] Errandonea, D., Burakovsky, L., Preston, D. L., MacLeod, S. G., Santamaría-Perez, D., Chen, S., Cynn, H., Simak, S. I., McMahon, M. I., Proctor, J. E. & Mezouar, M. (2020). *Commun. Mater.* **1**, 60.

[bb15] Falkenberg, G., Seiboth, F., Koch, F., Falch, K. V., Schropp, A., Brückner, D. & Garrevoet, J. (2020). *Powder Diffr.* **35**, S34–S37.

[bb16] Fedotenko, T., Dubrovinsky, L., Aprilis, G., Koemets, E., Snigirev, A., Snigireva, I., Barannikov, A., Ershov, P., Cova, F., Hanfland, M. & Dubrovinskaia, N. (2019). *Rev. Sci. Instrum.* **90**, 104501.

[bb17] Fei, Y., Ricolleau, A., Frank, M., Mibe, K., Shen, G. & Prakapenka, V. (2007). *Proc. Natl Acad. Sci. USA*, **104**, 9182–9186. 10.1073/pnas.0609013104 PMC1890468 17483460

[bb18] Fei, Y., Seagle, C. T., Townsend, J. P., McCoy, C. A., Boujibar, A., Driscoll, P., Shulenburger, L. & Furnish, M. D. (2021). *Nat. Commun.* **12**, 876. 10.1038/s41467-021-21170-y PMC7873221 33563984

[bb19] Friedrich, A., Collings, I. E., Dziubek, K. F., Fanetti, S., Radacki, K., Ruiz-Fuertes, J., Pellicer-Porres, J., Hanfland, M., Sieh, D., Bini, R., Clark, S. J. & Marder, T. B. (2020). *J. Am. Chem. Soc.* **142**, 18907–18923. 10.1021/jacs.0c09021 33095990

[bb20] Grosu, Y., Faik, A., Ortega-Fernández, I. & D’Aguanno, B. (2017). *Solar Energy Mater. Solar Cells*, **161**, 170–176.

[bb46] Hrubiak, R., Smith, J. S. & Shen, G. (2019). *Rev. Sci. Instrum.* **90**, 025109. 10.1063/1.5057518 30831723

[bb21] Huang, H. & Xu, J. Y. (2019). *Environ. Sci.* **267**, 042019.

[bb22] Jenei, Z., O’Bannon, E. F., Weir, S. T., Cynn, H., Lipp, M. J. & Evans, W. J. (2018). *Nat. Commun.* **9**, 3563. 10.1038/s41467-018-06071-x PMC6120914 30177697

[bb23] Kantor, I., Prakapenka, V., Kantor, A., Dera, P., Kurnosov, A., Sinogeikin, S., Dubrovinskaia, N. & Dubrovinsky, L. (2012). *Rev. Sci. Instrum.* **83**, 125102. 10.1063/1.4768541 23278021

[bb24] Kawamura, H., Akahama, Y., Umemoto, S., Takemura, K., Ohishi, Y. & Shimomura, O. (2002). *J. Phys. Condens. Matter*, **14**, 10407–10410.

[bb25] Khandarkhaeva, S., Fedotenko, T., Bykov, M., Bykova, E., Chariton, S., Sedmak, P., Glazyrin, K., Prakapenka, V., Dubrovinskaia, N. & Dubrovinsky, L. (2020). *Eur. J. Inorg. Chem.* **2020**, 2186–2190.

[bb26] Kurnosov, A., Kantor, I., Boffa-Ballaran, T., Lindhardt, S., Dubrovinsky, L., Kuznetsov, A. & Zehnder, B. H. (2008). *Rev. Sci. Instrum.* **79**, 045110. 10.1063/1.2902506 18447555

[bb27] Laniel, D., Winkler, B., Bykova, E., Fedotenko, T., Chariton, S., Milman, V., Bykov, M., Prakapenka, V., Dubrovinsky, L. & Dubrovinskaia, N. (2020). *Phys. Rev. B*, **102**, 134109. 10.1103/PhysRevLett.124.216001 32530671

[bb28] Lengeler, B., Schroer, C. G., Kuhlmann, M., Benner, B., Günzler, T. F., Kurapova, O., Zontone, F., Snigirev, A. & Snigireva, I. (2005). *J. Phys. D Appl. Phys.* **38**, A218–A222.

[bb29] Liermann, H.-P., Konôpková, Z., Morgenroth, W., Glazyrin, K., Bednarčik, J., McBride, E. E., Petitgirard, S., Delitz, J. T., Wendt, M., Bican, Y., Ehnes, A., Schwark, I., Rothkirch, A., Tischer, M., Heuer, J., Schulte-Schrepping, H., Kracht, T. & Franz, H. (2015). *J. Synchrotron Rad.* **22**, 908–924. 10.1107/S1600577515005937 PMC4489534 26134794

[bb30] Matos, M., Hoffmann, R., Latgé, A. & Anda, E. V. (1996). *Chem. Mater.* **8**, 2324–2330.

[bb31] McMahon, M. (2018). *Nat. Mater.* **17**, 858–859. 10.1038/s41563-018-0177-3 30250069

[bb32] Palmer, D. C. (2014). *CrystalMaker.* CrystalMaker Software Ltd, Begbroke, England.

[bb33] Petříček, V., Dušek, M. & Palatinus, L. (2006). *JANA2006.* Institute of Physics, Czech Academy of Sciences, Prague, Czech Republic.

[bb47] Petříček, V., Dušek, M. & Palatinus, L. (2014). *Z. Kristallogr. Cryst. Mater.* **229**, 345–352.

[bb48] Prescher, C. & Prakapenka, V. B. (2015). *High Press. Res.* **35**, 223–230.

[bb34] Rigaku (2019). *CrysAlis PRO*. Version 171.40. Rigaku Corporation, Wroclaw, Poland.

[bb49] Rigaku Oxford Diffraction (2020). *CrysAlis PRO*. Rigaku Oxford Diffraction, The Woodlands, Texas, USA.

[bb35] Schroer, C., Agapov, I., Roehlsberger, R., Wanzenberg, R., Brinkmann, R., Weckert, E. & Leemans, W. (2019). *PETRA IV: Upgrade of PETRA III to the Ultimate 3D X-ray Microscope, Conceptual Design Report (CDR)*. https://bib-pubdb1.desy.de/record/426140.

[bb36] Seiboth, F., Brückner, D., Kahnt, M., Lyubomirskiy, M., Wittwer, F., Dzhigaev, D., Ullsperger, T., Nolte, S., Koch, F., David, C., Garrevoet, J., Falkenberg, G. & Schroer, C. G. (2020). *J. Synchrotron Rad.* **27**, 1121–1130. 10.1107/S1600577520007900 PMC7467333 32876586

[bb37] Seiboth, F., Kahnt, M., Scholz, M., Seyrich, M., Wittwer, F., Garrevoet, J., Falkenberg, G., Schropp, A. & Schroer, C. G. (2016). *Proc. SPIE*, **9963**, 99630P.

[bb38] Seiboth, F., Schropp, A., Scholz, M., Wittwer, F., Rödel, C., Wünsche, M., Ullsperger, T., Nolte, S., Rahomäki, J., Parfeniukas, K., Giakoumidis, S., Vogt, U., Wagner, U., Rau, C., Boesenberg, U., Garrevoet, J., Falkenberg, G., Galtier, E. C., Ja Lee, H., Nagler, B. & Schroer, C. G. (2017). *Nat. Commun.* **8**, 14623. 10.1038/ncomms14623 PMC5337966 28248317

[bb39] Sheldrick, G. M. (2008). *Acta Cryst.* A**64**, 112–122. 10.1107/S0108767307043930 18156677

[bb40] Sheldrick, G. M. (2015). *Acta Cryst.* A**71**, 3–8.

[bb41] Tateno, S., Hirose, K., Ohishi, Y. & Tatsumi, Y. (2010). *Science*, **330**, 359–361. 10.1126/science.1194662 20947762

[bb42] Vasiukov, D. (2018). PhD thesis. University of Bayreuth, Germany.

[bb43] Wang, Y., Li, Q., Chen, N., Cheng, J.-M., Li, C.-G., Li, H., Long, Q.-H., Shi, J.-S. & Deng, J.-J. (2016). *Chin. Phys. C*, **40**, 086205.

