# Supplementary material for: Sub-micrometer focusing setup for high-pressure crystallography at the Extreme Conditions beamline at PETRA III
Source: J Synchrotron Radiat. 2022 Apr 4;29(Pt 3):654–63. doi: 10.1107/S1600577522002582 (PMC9070721; doi:10.1107/S1600577522002582)
Supplement: Supplementary file 3 [file s-29-00654-sup3.pdf]

JOURNAL OF SYNCHROTRON RADIATION

Volume 29 (2022)

Supporting information for article:

Sub-micrometre focusing setup for high-pressure crystallography on the Extreme Conditions Beamline at PETRA III

K. Glazyrin, S. Khandarkhaeva, T. Fedotenko, W. Dong, D. Laniel, F. Seiboth, A. Schropp, J. Garrevoet, D. Brückner, G. Falkenberg, A. Kubec, C. David, M. Wendt, S. Wenz, L. Dubrovinsky, Natalia Dubrovinskaia and H.-P. Liermann

Supplementary

Sub-micron focusing setup for high-pressure crystallography at the Extreme Conditions Beamline of PETRA III

K. Glazyrin[1], S. Khandarkhaeva[2,3], T. Fedotenko[1,3], W. Dong[1], D. Laniel[3], F. Seiboth[4], A. Schropp[4,5], J. Garrevoet[1], D. Brückner[1,6,7], G. Falkenberg[1], A. Kubec[8,9], C. David[8], M. Wendt[1], S. Wenz[1], L. Dubrovinsky[2], N. Dubrovinskaia[3,10], H.-P. Liermann[1]

[1]Deutsches Elektronen-Synchrotron DESY, Notkestr. 85, 22607 Hamburg, Germany

[2]Bayerisches Geoinstitut, University of Bayreuth, Universitätsstr. 30, 95440 Bayreuth, Germany

[3]Material Physics and Technology at Extreme Conditions, Laboratory of Crystallography, University of Bayreuth, Universitätsstr. 30, 95440 Bayreuth, Germany

[4]CXNS - Center for X-ray and Nano Science, Deutsches Elektronen Synchrotron DESY, Notkestr. 85, 22607 Hamburg, Germany

[5]Helmholtz Imaging Platform, Deutsches Elektronen-Synchrotron DESY, Notkestraße 85, 22607 Hamburg, Germany

[6]Department Physik, Universität Hamburg, Luruper Chaussee 149, 22761 Hamburg, Germany

[7]Ruhr-Universität Bochum, Universitätsstr. 150, 44801 Bochum, Germany

[8]Laboratory for Micro- and Nanotechnology, Paul Scherrer Institut, Forschungsstr. 111 5232 Villigen-PSI, Switzerland

[9]Current affiliation: XRnanotech GmbH, Forschungsstr. 111, 5232 Villigen-PSI, Switzerland

[10]Department of Physics, Chemistry and Biology (IFM), Linköping University, Campus Valla, Fysikhuset, F310, SE-581 83, Linköping, Sweden

Procedure for the analysis of the multigrain single-crystal X-ray diffraction dataset collected in a laser-heated diamond anvil cell (*oP*-Fe_3O_4 example):

1) Prior to collection of SCXRD datasets, a 2D X-ray mapping of still images (without sample rotation) was performed over the heated area in order to find the best position for the data acquisition. The most intense signal of the phase was identified during manual inspection of collected map in DIOPTAS (Prescher & Prakapenka, 2015) and XDI (Hrubiak *et al.*, 2019) software packages (**Fig.1**). In both cases, the corresponding instrumental model was calibrated using CeO_2 powder standard from NIST (674b).

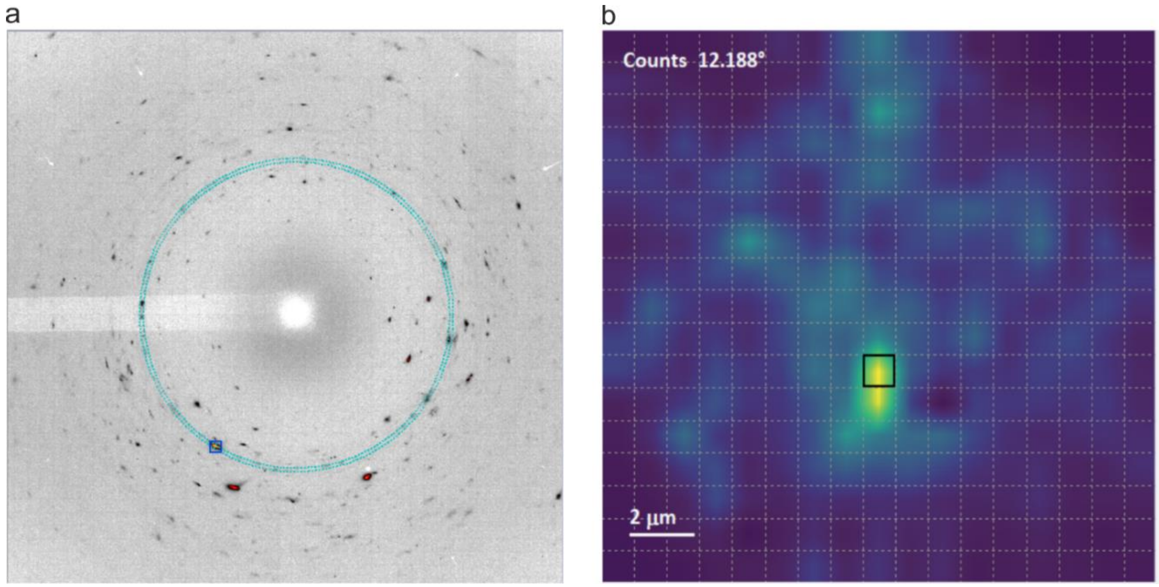

Fig.1. Analysis of 2D X-ray map indicates the position of the phase of interest in the pressure chamber. (a) A diffraction image, revealing the presence of single crystalline grain, suitable for further studies. Its position within the 2D grid is indicated in (b) where we show a reconstructed X-ray image of the sample chamber (17×17 μm^2, 1 µm step size), showing the position, where diffraction peak signal of the new phase (later identified as *oP*-Fe_3O_4) were the most intense. The map suggests that the grain was elongated in the vertical direction.

2) Single-crystal XRD datasets were collected via rotation around vertical ω-axis from -38˚ to 38˚, with the angular step of 0.5˚ and the acquisition time of 10 seconds/frame. During the data analysis, a reconstruction of the peak table in the reciprocal space was prepared (**Fig. 2a**) using CrysAlisPro software (Rigaku Oxford Diffraction, 2020). A crystal of orthoenstatite $(Mg_{1.93},Fe_{0.06})(Si_{1.93},Al_{0.06})O_6$ (space group *Pbca*, a=8.8117(2) Å, b=5.18320(10) Å, c=18.2391(3) Å) was used as a calibration standard for refinement of the CrysAlisPro instrumental parameters of the "sub-micron" setup (the sample-to-detector distance, the detector's origin, offsets of the

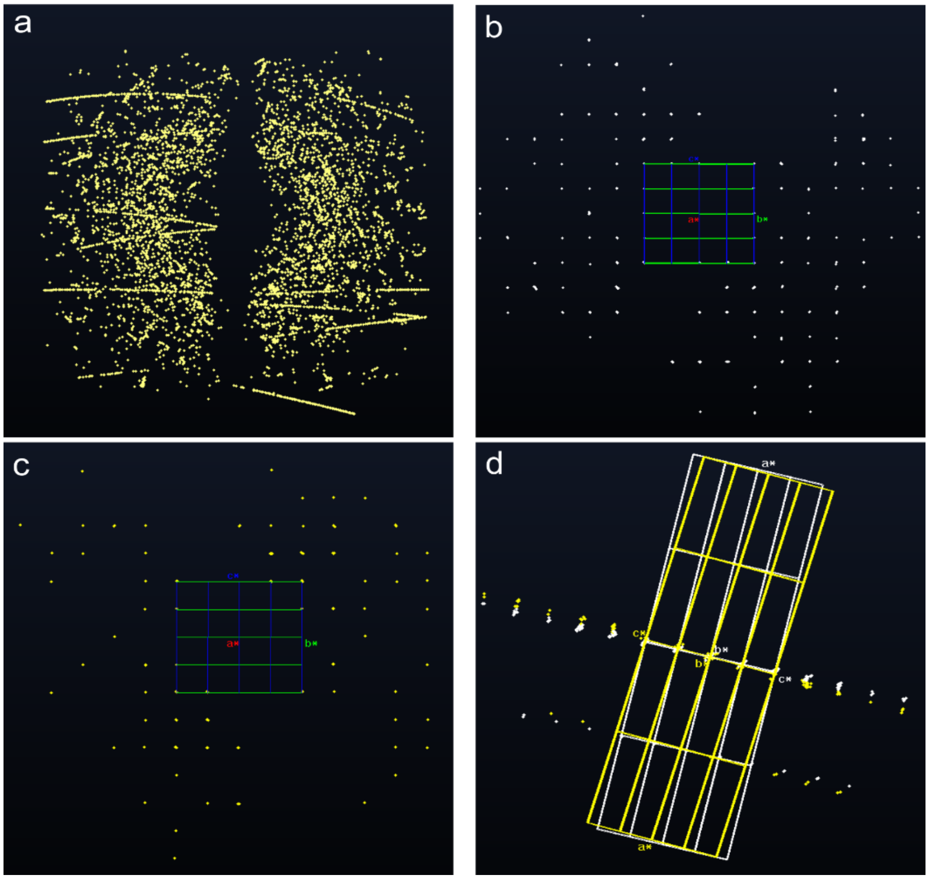

Fig. 2. The results of the peak search reconstruction and indexing in the reciprocal space using CrysAlisPro for the case of laser heated hematite. (a) 3D representation of the detected scattering signal coming from the best-spot probed area. *If the sample would be illuminated by a beam of a larger area, the reciprocal space would be even more densely populated increasing the difficulty of the analysis tenfold*. If we focus on *oP*-Fe_3O_4, in (b) and (c) we show only the signal corresponding to single-crystalline domains of *oP*-Fe_3O_4 found in data, and we illustrate the relation between them (a misorientation around b*-axis) in the panel (d). We integrated the strongest scattering domains using masking of their strong overlapping reflections.

goniometer angles and rotations of the X-ray beam and the detector around the instrument axis, etc.).

3) Conventionally for a DAC experiment, the peak search table contains peaks from all crystalline phases, diffuse scattering and even detector related artifacts, e.g. originate from over saturated diffraction spot (**Fig.2a**). Detected intensity can be identified as contributions from reaction products, initial reagents, pressure-transmitting medium, diamonds, gasket material, etc. Manual

inspection of the 3D reciprocal space in CryAlisPro allows a separation of reciprocal lattices of the well-diffracting domains representing the phase of interest (**Fig. 2b, c**).

4) In our study of Fe_3O_4, the indexation and refinement of the unit cell parameters reveal 265 peaks and 185 peaks found for domain #1 and domain #2, respectively. Once the unit cell parameters were defined and orientation matrixes of the two observed domains were determined - the integrated sample reflection intensities were extracted from the collect images. During the conventional data processing workflow, after data integration, CrysAlisPro applies frame scaling, absorption corrections and allows for a more careful inspection of the sample space group, providing statistics of the possible systematic absences, etc. The statistics is correlated with a great number of parameters controlling integration: correct frame ranges, DAC's opening angle, integration box size, reflection profile fitting and background evaluation schemes, etc. It requires a careful approach and inspection at various steps of the iterative analysis. For example, we always inspect intensity and resolution statistics in order to confirm consistency between intensities of equivalent reflections (if possible), degree of peak oversaturation, etc. We also pay close attention to R-factors, namely R_σ, $F^2_{obs}/\sigma_{int}(F^2_{obs})$, R_{int} and to the frame-by-frame scaling coefficients (e.g. empirical absorption correction). The values of crystallographic R-factors are generally accepted indicators of the sample and data quality. The parameters corresponding to the best crystal structure solutions are provided in the manuscript.

Considering the example of *oP*-Fe_3O_4, we note that each of the strongly scattering domains turned out to be a single crystalline grain. Based on the number of observable reflections, we mark the domain #1 as "the single crystalline grain of the best quality". Indeed, its R_{int} =2.9% is much lower if compared to domain #2 with R_{int} > 15%. The structure of domain #1 was solved with the SHELXT (Sheldrick, 2015) structure solution program using intrinsic phasing which was refined with the JANA2006 program (Petříček *et al.*, 2014). Unfortunately, it was not possible to solve and refine the structure of domain #2 independently. However, similar lattice parameters and indexing with a supplementary inspection of the reciprocal space slices (e.g. *hk0, h0l, 0kl*) clearly indicate a differently oriented grain of *oP*-Fe_3O_4.

The analysis of the $Mg_{0.91(2)}Fe_{0.09(2)}SiO_3$ was performed in the similar fashion.

For additional information about treatment of SCXRD data, collected in DACs we kindly direct to the well-illustrated publication of (Bykova, 2015).

References

Bykova, E. PhD Thesis (2015). Single-crystal X-ray diffraction at extreme conditions in mineral physics and material sciences. University of Bayreuth. https://epub.uni-bayreuth.de/2124/

Hrubiak, R., Smith, J. S. & Shen, G. (2019). *Rev. Sci. Instrum.* **90**, 025109.

Petříček, V., Dušek, M. & Palatinus, L. (2014). *Zeitschrift Für Krist. - Cryst. Mater.* **229**, 345–352.

Prescher, C. & Prakapenka, V. B. (2015). *High Press. Res.* **35**, 223–230.

Rigaku Oxford Diffraction (2020).

Sheldrick, G. M. (2015). *Acta Crystallogr. Sect. A Found. Adv.* **71**, 3–8.
