# Supplementary material for: Sub-micrometer focusing setup for high-pressure crystallography at the Extreme Conditions beamline at PETRA III
Source: J Synchrotron Radiat. 2022 Apr 4;29(Pt 3):654–63. doi: 10.1107/S1600577522002582 (PMC9070721; doi:10.1107/S1600577522002582)

checkCIF/PLATON report

Structure factors have been supplied for datablock(s) I

THIS REPORT IS FOR GUIDANCE ONLY. IF USED AS PART OF A REVIEW PROCEDURE FOR PUBLICATION, IT SHOULD NOT REPLACE THE EXPERTISE OF AN EXPERIENCED CRYSTALLOGRAPHIC REFEREE.

No syntax errors found. CIF dictionary Interpreting this report

Datablock: I

Bond precision: Mg- O = 0.0030 A Wavelength=0.48300

Cell:	a=5.1815(2)	b=18.2321(11)	c=8.8085(5)
	alpha=90	beta=90	gamma=90
Temperature:	293 K		

	Calculated	Reported
Volume	832.14(8)	832.14(8)
Space group	P b c a	P b c a
Hall group	-P 2ac 2ab	-P -2xab;-2
Moiety formula	Al0.54 Fe0.54 Mg15.46 O48 Si15.46	Mg1.94 Fe0.067 Si1.93 Al0.067 O6
Sum formula	Al0.54 Fe0.54 Mg15.46 O48 Si15.46	Mg1.94 Fe0.067 Si1.93 Al0.067 O6
Mr	1622.71	202.90
Dx,g cm-3	3.238	3.238
Z	1	8
Mu (mm-1)	0.448	0.436
F000	807.0	807.0
F000’	807.94	
h,k,lmax	6,23,11	6,19,9
Nref	971	454
Tmin,Tmax		0.824,1.000
Tmin’		

Correction method= # Reported T Limits: Tmin=0.824 Tmax=1.000
AbsCorr = MULTI-SCAN

Data completeness= 0.468 Theta(max)= 18.360

R(reflections)= 0.0253(409) wR2(reflections)= wR= 0.0391(454)

S = 1.870 Npar= 61

```
The following ALERTS were generated. Each ALERT has the format
       test-name_ALERT_alert-type_alert-level.
Click on the hyperlinks for more details of the test.
```

Alert level A

```
PLAT027_ALERT_3_A _diffrn_reflns_theta_full value (too) Low ......      12.67 Degree
```

Author Response: The dataset was incomplete since the data were collected in a diamond anvil cell metallic body of which shadows more than 60% of the reflections

```
PLAT029_ALERT_3_A _diffrn_measured_fraction_theta_full value Low .      0.730 Why?
```

Author Response: The dataset was incomplete since the data were collected in a diamond anvil cell metallic body of which shadows more than 60% of the reflections.

Alert level B

```
PLAT088_ALERT_3_B Poor Data / Parameter Ratio ....................       7.44 Note
              O1        O2        O3        O4        O5                  etc.
```

Author Response: Poor data/parameter ratio is due to big amount of the parameters to refine and incompleteness of the dataset (measurement in a diamond anvil cell).

Alert level C

```
PLAT041_ALERT_1_C Calc. and Reported SumFormula    Strings  Differ     Please Check
PLAT077_ALERT_4_C Unitcell Contains Non-integer Number of Atoms ..     Please Check
PLAT127_ALERT_1_C Implicit Hall Symbol  Inconsistent with Explicit  -P -2xa  b;-2ybc
PLAT220_ALERT_2_C NonSolvent   Resd 1 Mg   Ueq(max)/Ueq(min) Range        3.3 Ratio
PLAT918_ALERT_3_C Reflection(s) with I(obs) much Smaller I(calc) .          1 Check
PLAT939_ALERT_3_C Large Value of Not (SHELXL) Weight Optimized S .      38.63 Check
PLAT975_ALERT_2_C Check Calcd Resid. Dens.  0.62A   From O1            0.51 eA-3
PLAT975_ALERT_2_C Check Calcd Resid. Dens.  0.50A   From O3            0.43 eA-3
PLAT975_ALERT_2_C Check Calcd Resid. Dens.  1.00A   From O6            0.42 eA-3
PLAT976_ALERT_2_C Check Calcd Resid. Dens.  0.97A   From O1           -0.44 eA-3
```

Alert level G

```
ABSMU01_ALERT_1_G  Calculation of _exptl_absorpt_correction_mu
                not performed for this radiation type.
CELLZ01_ALERT_1_G Difference between formula and atom_site contents detected.
CELLZ01_ALERT_1_G ALERT: check formula stoichiometry or atom site occupancies.
           From the CIF: _cell_formula_units_Z    8
           From the CIF: _chemical_formula_sum  Mg1.94 Fe0.067 Si1.93 Al0.067 O6
           TEST: Compare cell contents of formula and atom_site data

           atom    Z*formula  cif sites diff
           Mg        15.52     15.46    0.06
           Fe         0.54      0.54    0.00
           Si        15.44     15.46   -0.02
```

```
          Al         0.54      0.54    0.00
          O         48.00     48.00    0.00
PLAT004_ALERT_5_G Polymeric Structure Found with Maximum Dimension          3 Info
PLAT005_ALERT_5_G No Embedded Refinement Details Found  in the CIF     Please Do !
PLAT042_ALERT_1_G Calc. and Reported MoietyFormula Strings  Differ     Please Check
PLAT045_ALERT_1_G Calculated and Reported Z Differ by a Factor ...       0.13 Check
PLAT092_ALERT_4_G Check: Wavelength Given is not Cu,Ga,Mo,Ag,In Ka    0.48300 Ang.
PLAT199_ALERT_1_G Reported _cell_measurement_temperature ..... (K)        293 Check
PLAT200_ALERT_1_G Reported   _diffrn_ambient_temperature ..... (K)        293 Check
PLAT300_ALERT_4_G Atom Site Occupancy of Fe3       Constrained at      0.067 Check
PLAT300_ALERT_4_G Atom Site Occupancy of Si2       Constrained at      0.933 Check
PLAT300_ALERT_4_G Atom Site Occupancy of Mg3       Constrained at      0.933 Check
PLAT300_ALERT_4_G Atom Site Occupancy of Al2       Constrained at      0.067 Check
PLAT301_ALERT_3_G Main Residue  Disorder .............(Resd  1  )        20% Note
PLAT808_ALERT_5_G No Parseable SHELXL Style Weighting Scheme Found     Please Check
PLAT883_ALERT_1_G No Info/Value for _atom_sites_solution_primary .     Please Do !
PLAT910_ALERT_3_G Missing # of FCF Reflection(s) Below Theta(Min).          1 Note
PLAT911_ALERT_3_G Missing FCF Refl Between Thmin & STh/L=    0.600        300 Report
PLAT912_ALERT_4_G Missing # of FCF Reflections Above STh/L=  0.600        129 Note
PLAT913_ALERT_3_G Missing # of Very Strong Reflections in FCF ....          3 Note
PLAT929_ALERT_5_G No Weight Pars,Obs and Calc R1,wR2,S not Checked          ! Info
PLAT951_ALERT_5_G Calculated (ThMax) and CIF-Reported Kmax Differ           4 Units
PLAT952_ALERT_5_G Calculated (ThMax) and CIF-Reported Lmax Differ           2 Units
PLAT957_ALERT_1_G Calculated (ThMax) and Actual (FCF) Kmax Differ           4 Units
PLAT958_ALERT_1_G Calculated (ThMax) and Actual (FCF) Lmax Differ           2 Units
PLAT960_ALERT_3_G Number of Intensities with I  <   - 2*sig(I) ...          1 Check
PLAT985_ALERT_1_G The Fe-f"=    0.4161 Deviates from the B&C-Value     0.4134 Check
```

```
   2 ALERT level A = Most likely a serious problem - resolve or explain
   1 ALERT level B = A potentially serious problem, consider carefully
  10 ALERT level C = Check. Ensure it is not caused by an omission or oversight
  28 ALERT level G = General information/check it is not something unexpected

  13 ALERT type 1 CIF construction/syntax error, inconsistent or missing data
   5 ALERT type 2 Indicator that the structure model may be wrong or deficient
  10 ALERT type 3 Indicator that the structure quality may be low
   7 ALERT type 4 Improvement, methodology, query or suggestion
   6 ALERT type 5 Informative message, check
```

It is advisable to attempt to resolve as many as possible of the alerts in all categories. Often the minor alerts point to easily fixed oversights, errors and omissions in your CIF or refinement strategy, so attention to these fine details can be worthwhile. In order to resolve some of the more serious problems it may be necessary to carry out additional measurements or structure refinements. However, the purpose of your study may justify the reported deviations and the more serious of these should normally be commented upon in the discussion or experimental section of a paper or in the "special_details" fields of the CIF. checkCIF was carefully designed to identify outliers and unusual parameters, but every test has its limitations and alerts that are not important in a particular case may appear. Conversely, the absence of alerts does not guarantee there are no aspects of the results needing attention. It is up to the individual to critically assess their own results and, if necessary, seek expert advice.

Publication of your CIF in IUCr journals

A basic structural check has been run on your CIF. These basic checks will be run on all CIFs submitted for publication in IUCr journals (*Acta Crystallographica*, *Journal of Applied Crystallography*, *Journal of Synchrotron Radiation*); however, if you intend to submit to *Acta Crystallographica Section C* or *E* or *IUCrData*, you should make sure that full publication checks are run on the final version of your CIF prior to submission.

Publication of your CIF in other journals

Please refer to the *Notes for Authors* of the relevant journal for any special instructions relating to CIF submission.

PLATON version of 05/12/2020; check.def file version of 05/12/2020

Datablock I - ellipsoid plot

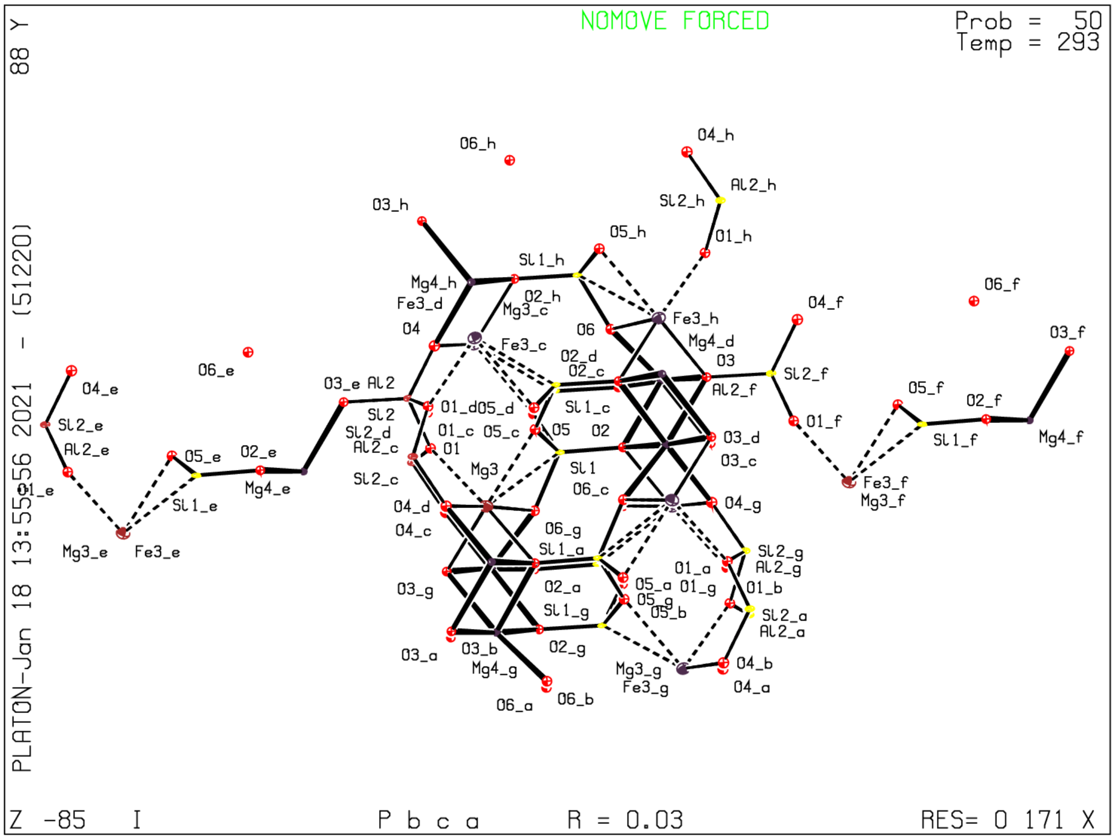

Supplement: Supplementary file 4 [file s-29-00654-sup4.zip › 01_Enst_nano_0GPa/checkcif_Table2-Enstatite-tiny.pdf]
