# Supplementary material for: Sub-micrometer focusing setup for high-pressure crystallography at the Extreme Conditions beamline at PETRA III
Source: J Synchrotron Radiat. 2022 Apr 4;29(Pt 3):654–63. doi: 10.1107/S1600577522002582 (PMC9070721; doi:10.1107/S1600577522002582)

checkCIF/PLATON report

Structure factors have been supplied for datablock(s) I

THIS REPORT IS FOR GUIDANCE ONLY. IF USED AS PART OF A REVIEW PROCEDURE FOR PUBLICATION, IT SHOULD NOT REPLACE THE EXPERTISE OF AN EXPERIENCED CRYSTALLOGRAPHIC REFEREE.

No syntax errors found.   CIF dictionary   Interpreting this report

Datablock: I

```
Bond precision:  Sb-Sb = 0.0006 A        Wavelength=0.48300

Cell:              a=9.0360(1)      b=9.0360(1)      c=9.0360(1)
                   alpha=90         beta=90          gamma=90
Temperature:       293 K

                Calculated                 Reported
Volume          737.78(2)                  737.783(14)
Space group     I m -3                     I m -3
Hall group      -I 2 2 3                   -I 2;2;3
Moiety formula  Co Sb3                     Co1 Sb3
Sum formula     Co Sb3                     Co1 Sb3
Mr              424.21                     424.20
Dx,g cm-3       7.638                      7.640
Z               8                          8
Mu (mm-1)       8.950                      8.830
F000            1440.0                     1440.0
F000'           1406.45
h,k,lmax        11,11,11                   7,10,10
Nref            168                        133
Tmin,Tmax                                  0.888,1.000
Tmin'

Correction method= # Reported T Limits: Tmin=0.888 Tmax=1.000
AbsCorr = MULTI-SCAN

Data completeness= 0.792          Theta(max)= 18.160

R(reflections)= 0.0121( 121)      wR2(reflections)= 0.0431( 133)

S = 1.340                  Npar= 9
```

The following ALERTS were generated. Each ALERT has the format
test-name_ALERT_alert-type_alert-level.
Click on the hyperlinks for more details of the test.

Alert level A

```
PLAT027_ALERT_3_A _diffrn_reflns_theta_full value (too) Low ......      15.50 Degree
```

Author Response: The dataset was incomplete since the data were collected in a diamond anvil cell metallic body of which shadows more than 60% of the reflections

---

Alert level B

```
PLAT911_ALERT_3_B Missing FCF Refl Between Thmin & STh/L=    0.600         13 Report
```

Author Response: Certain part of the reflections is missing due to geometry of the experiment. The metallic body of the diamond anvil cell absorbs more than 60% of the reflections.

Alert level C

```
PLAT127_ALERT_1_C Implicit Hall Symbol  Inconsistent with Explicit  -I 2;2;  3
PLAT971_ALERT_2_C Check Calcd Resid. Dens.  2.53A   From  Co1            1.62 eA-3
```

Alert level G

```
ABSMU01_ALERT_1_G  Calculation of _exptl_absorpt_correction_mu
                 not performed for this radiation type.
PLAT004_ALERT_5_G Polymeric Structure Found with Maximum Dimension          1 Info
PLAT005_ALERT_5_G No Embedded Refinement Details Found  in the CIF     Please Do !
PLAT092_ALERT_4_G Check: Wavelength Given is not Cu,Ga,Mo,Ag,In Ka    0.48300 Ang.
PLAT152_ALERT_1_G The Supplied and Calc. Volume s.u. Differ by ...          6 Units
PLAT199_ALERT_1_G Reported _cell_measurement_temperature ..... (K)        293 Check
PLAT200_ALERT_1_G Reported   _diffrn_ambient_temperature ..... (K)        293 Check
PLAT883_ALERT_1_G No Info/Value for _atom_sites_solution_primary .     Please Do !
PLAT912_ALERT_4_G Missing # of FCF Reflections Above STh/L=  0.600         15 Note
PLAT950_ALERT_5_G Calculated (ThMax) and CIF-Reported Hmax Differ           4 Units
PLAT984_ALERT_1_G The Sb-f'=   -1.4957 Deviates from the B&C-Value    -1.4882 Check
PLAT985_ALERT_1_G The Co-f"=    0.4817 Deviates from the B&C-Value     0.4788 Check
PLAT985_ALERT_1_G The Sb-f"=    0.7841 Deviates from the B&C-Value     0.7746 Check
```

```
   1 ALERT level A = Most likely a serious problem - resolve or explain
   1 ALERT level B = A potentially serious problem, consider carefully
   2 ALERT level C = Check. Ensure it is not caused by an omission or oversight
  13 ALERT level G = General information/check it is not something unexpected

   9 ALERT type 1 CIF construction/syntax error, inconsistent or missing data
   1 ALERT type 2 Indicator that the structure model may be wrong or deficient
   2 ALERT type 3 Indicator that the structure quality may be low
   2 ALERT type 4 Improvement, methodology, query or suggestion
   3 ALERT type 5 Informative message, check
```

It is advisable to attempt to resolve as many as possible of the alerts in all categories. Often the minor alerts point to easily fixed oversights, errors and omissions in your CIF or refinement strategy, so attention to these fine details can be worthwhile. In order to resolve some of the more serious problems it may be necessary to carry out additional measurements or structure refinements. However, the purpose of your study may justify the reported deviations and the more serious of these should normally be commented upon in the discussion or experimental section of a paper or in the "special_details" fields of the CIF. checkCIF was carefully designed to identify outliers and unusual parameters, but every test has its limitations and alerts that are not important in a particular case may appear. Conversely, the absence of alerts does not guarantee there are no aspects of the results needing attention. It is up to the individual to critically assess their own results and, if necessary, seek expert advice.

Publication of your CIF in IUCr journals

A basic structural check has been run on your CIF. These basic checks will be run on all CIFs submitted for publication in IUCr journals (*Acta Crystallographica*, *Journal of Applied Crystallography*, *Journal of Synchrotron Radiation*); however, if you intend to submit to *Acta Crystallographica Section C* or *E* or *IUCrData*, you should make sure that full publication checks are run on the final version of your CIF prior to submission.

Publication of your CIF in other journals

Please refer to the *Notes for Authors* of the relevant journal for any special instructions relating to CIF submission.

PLATON version of 05/12/2020; check.def file version of 05/12/2020

Datablock I - ellipsoid plot

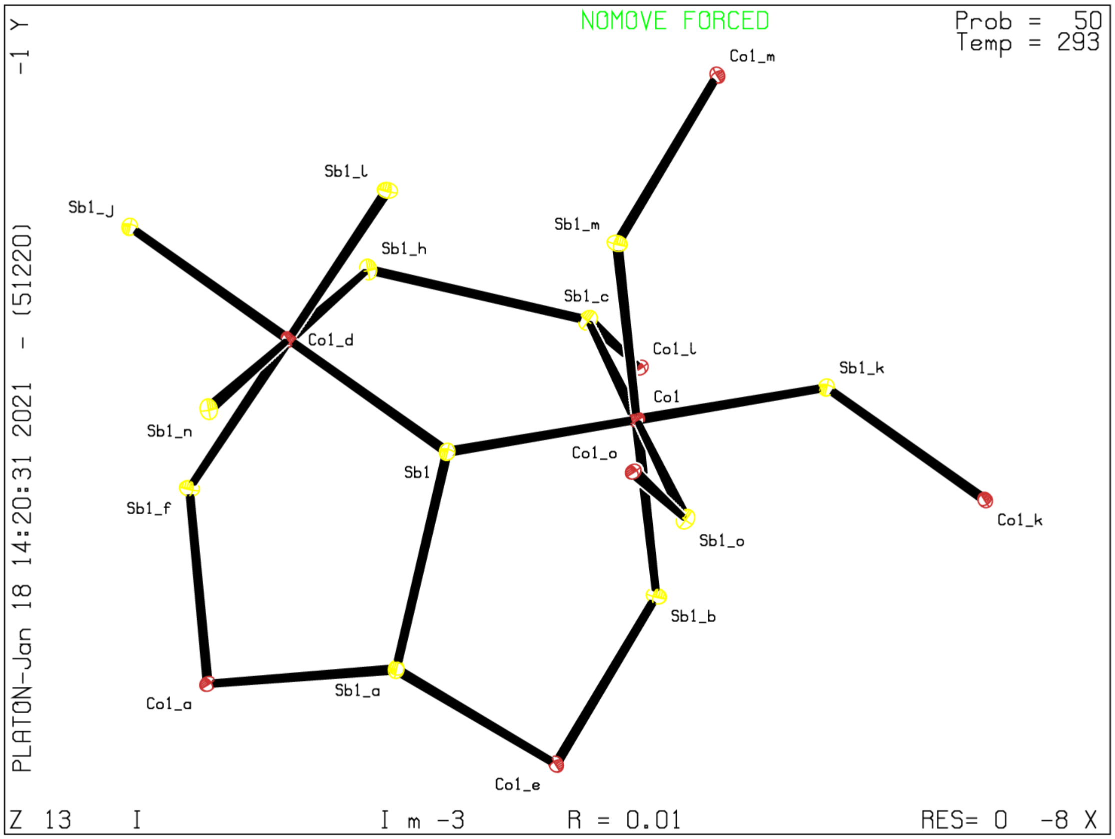

Supplement: Supplementary file 4 [file Table2-CoSb3-tiny.pdf › 02_CoSb3_0GPa/Table2-CoSb3-tiny.pdf]
