# Supplementary material for: Sub-micrometer focusing setup for high-pressure crystallography at the Extreme Conditions beamline at PETRA III
Source: J Synchrotron Radiat. 2022 Apr 4;29(Pt 3):654–63. doi: 10.1107/S1600577522002582 (PMC9070721; doi:10.1107/S1600577522002582)

checkCIF/PLATON report

Structure factors have been supplied for datablock(s) I

THIS REPORT IS FOR GUIDANCE ONLY. IF USED AS PART OF A REVIEW PROCEDURE FOR PUBLICATION, IT SHOULD NOT REPLACE THE EXPERTISE OF AN EXPERIENCED CRYSTALLOGRAPHIC REFEREE.

No syntax errors found.   CIF dictionary   Interpreting this report

Datablock: I

```
Bond precision:  Fe- O = 0.0221 A        Wavelength=0.48300

Cell:             a=7.932(10)     b=2.5881(13)     c=8.321(3)
                  alpha=90        beta=90          gamma=90
Temperature:      293 K

                Calculated                Reported
Volume          170.8(2)                  170.8(2)
Space group     P n m a                   P n m a
Hall group      -P 2ac 2n                 -P -2xabc;-
Moiety formula  Fe3 O4                    Fe3 O4
Sum formula     Fe3 O4                    Fe3 O4
Mr              231.55                    231.50
Dx,g cm-3       9.005                     9.002
Z               4                         4
Mu (mm-1)       8.346                     7.968
F000            440.0                     440.0
F000’           442.92
h,k,lmax        10,3,10                   7,3,9
Nref            234                       105
Tmin,Tmax                                 0.559,1.000
Tmin’

Correction method= # Reported T Limits: Tmin=0.559 Tmax=1.000
AbsCorr = MULTI-SCAN

Data completeness= 0.449          Theta(max)= 18.100

R(reflections)= 0.0708( 88)       wR2(reflections)= wR= 0.0751( 105)

S = 3.700                 Npar= 22
```

The following ALERTS were generated. Each ALERT has the format
test-name_ALERT_alert-type_alert-level.
Click on the hyperlinks for more details of the test.

Alert level A

```
ATOM007_ALERT_1_A  _atom_site_aniso_label is missing
            Unique label identifying the atom site.
```

Author Response: Due to incompleteness of the dataset thermal parameters were refined in isotropic approximation.

```
PLAT027_ALERT_3_A _diffrn_reflns_theta_full value (too) Low ......       9.38 Degree
```

Author Response: The dataset was incomplete since the data were collected in a diamond anvil cell metallic body of which shadows more than 60% of the reflections

```
PLAT029_ALERT_3_A _diffrn_measured_fraction_theta_full value Low .      0.700 Why?
```

Author Response: The dataset was incomplete since the data were collected in a diamond anvil cell metallic body of which shadows more than 60% of the reflections.

```
PLAT088_ALERT_3_A Poor Data / Parameter Ratio ...................       4.77 Note
```

Author Response: Poor data/parameter ratio is due big amount of the parameters to refine and incompleteness of the dataset (measurement in a diamond anvil cell).

Alert level C

```
DIFMX02_ALERT_1_C  The maximum difference density is > 0.1*ZMAX*0.75
            The relevant atom site should be identified.
GOODF01_ALERT_2_C  The least squares goodness of fit parameter lies
            outside the range 0.80 <> 2.00
            Goodness of fit given =      3.700
PLAT097_ALERT_2_C Large Reported Max.  (Positive) Residual Density       2.04 eA-3
PLAT127_ALERT_1_C Implicit Hall Symbol  Inconsistent with Explicit  -P -2xa  bc;-2yb
PLAT148_ALERT_3_C s.u. on the      a    - Axis is (Too) Large ....      0.010 Ang.
PLAT971_ALERT_2_C Check Calcd Resid. Dens.  0.87A   From O2             1.96 eA-3
PLAT971_ALERT_2_C Check Calcd Resid. Dens.  1.44A   From O1             1.68 eA-3
PLAT971_ALERT_2_C Check Calcd Resid. Dens.  0.87A   From Fe3            1.67 eA-3
PLAT971_ALERT_2_C Check Calcd Resid. Dens.  0.54A   From Fe1            1.63 eA-3
PLAT972_ALERT_2_C Check Calcd Resid. Dens.  0.92A   From O4            -1.63 eA-3
PLAT972_ALERT_2_C Check Calcd Resid. Dens.  0.82A   From Fe3           -1.54 eA-3
```

Alert level G

```
ABSMU01_ALERT_1_G  Calculation of _exptl_absorpt_correction_mu
                not performed for this radiation type.
PLAT004_ALERT_5_G Polymeric Structure Found with Maximum Dimension          3 Info
PLAT005_ALERT_5_G No Embedded Refinement Details Found  in the CIF     Please Do !
```

```
PLAT092_ALERT_4_G Check: Wavelength Given is not Cu,Ga,Mo,Ag,In Ka    0.48300 Ang.
PLAT199_ALERT_1_G Reported _cell_measurement_temperature ..... (K)        293 Check
PLAT200_ALERT_1_G Reported   _diffrn_ambient_temperature ..... (K)        293 Check
PLAT808_ALERT_5_G No Parseable SHELXL Style Weighting Scheme Found     Please Check
PLAT883_ALERT_1_G No Info/Value for _atom_sites_solution_primary .     Please Do !
PLAT910_ALERT_3_G Missing # of FCF Reflection(s) Below Theta(Min).          3 Note
PLAT911_ALERT_3_G Missing FCF Refl Between Thmin & STh/L=    0.600         70 Report
PLAT912_ALERT_4_G Missing # of FCF Reflections Above STh/L=  0.600         26 Note
PLAT913_ALERT_3_G Missing # of Very Strong Reflections in FCF ....          1 Note
PLAT929_ALERT_5_G No Weight Pars,Obs and Calc R1,wR2,S not Checked          ! Info
PLAT950_ALERT_5_G Calculated (ThMax) and CIF-Reported Hmax Differ           3 Units
PLAT956_ALERT_1_G Calculated (ThMax) and Actual (FCF) Hmax Differ           3 Units
PLAT985_ALERT_1_G The Fe-f"=    0.4161 Deviates from the B&C-Value     0.4134 Check
```

```
  4 ALERT level A = Most likely a serious problem - resolve or explain
  0 ALERT level B = A potentially serious problem, consider carefully
 11 ALERT level C = Check. Ensure it is not caused by an omission or oversight
 16 ALERT level G = General information/check it is not something unexpected

  9 ALERT type 1 CIF construction/syntax error, inconsistent or missing data
  8 ALERT type 2 Indicator that the structure model may be wrong or deficient
  7 ALERT type 3 Indicator that the structure quality may be low
  2 ALERT type 4 Improvement, methodology, query or suggestion
  5 ALERT type 5 Informative message, check
```

It is advisable to attempt to resolve as many as possible of the alerts in all categories. Often the minor alerts point to easily fixed oversights, errors and omissions in your CIF or refinement strategy, so attention to these fine details can be worthwhile. In order to resolve some of the more serious problems it may be necessary to carry out additional measurements or structure refinements. However, the purpose of your study may justify the reported deviations and the more serious of these should normally be commented upon in the discussion or experimental section of a paper or in the "special_details" fields of the CIF. checkCIF was carefully designed to identify outliers and unusual parameters, but every test has its limitations and alerts that are not important in a particular case may appear. Conversely, the absence of alerts does not guarantee there are no aspects of the results needing attention. It is up to the individual to critically assess their own results and, if necessary, seek expert advice.

Publication of your CIF in IUCr journals

A basic structural check has been run on your CIF. These basic checks will be run on all CIFs submitted for publication in IUCr journals (*Acta Crystallographica*, *Journal of Applied Crystallography*, *Journal of Synchrotron Radiation*); however, if you intend to submit to *Acta Crystallographica Section C* or *E* or *IUCrData*, you should make sure that full publication checks are run on the final version of your CIF prior to submission.

Publication of your CIF in other journals

Please refer to the *Notes for Authors* of the relevant journal for any special instructions relating to CIF submission.

PLATON version of 05/12/2020; check.def file version of 05/12/2020

Datablock I - ellipsoid plot

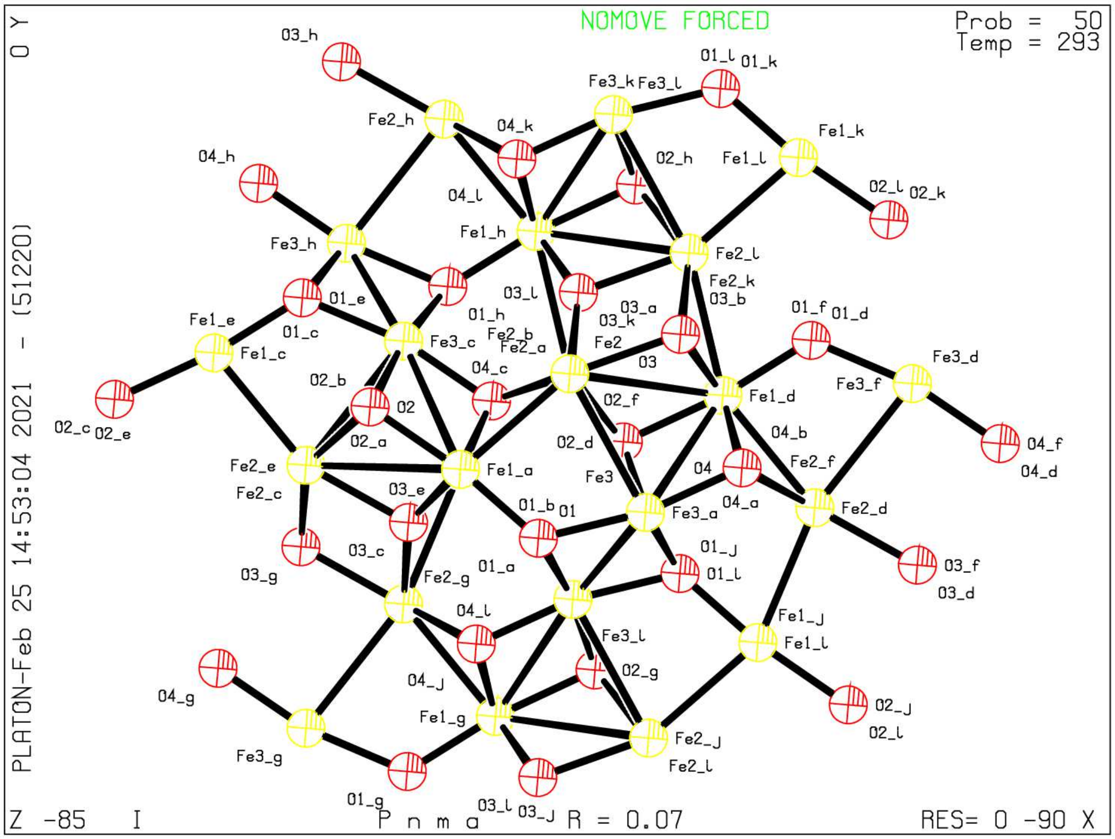

Supplement: Supplementary file 4 [file checkcif_Table3-Fe3O4_200 GPa.pdf › 03_oP-Fe3O4_200GPa/checkcif_Table3-Fe3O4_200 GPa.pdf]
