# Supplementary material for: Sub-micrometer focusing setup for high-pressure crystallography at the Extreme Conditions beamline at PETRA III
Source: J Synchrotron Radiat. 2022 Apr 4;29(Pt 3):654–63. doi: 10.1107/S1600577522002582 (PMC9070721; doi:10.1107/S1600577522002582)

checkCIF/PLATON report

Structure factors have been supplied for datablock(s) I

THIS REPORT IS FOR GUIDANCE ONLY. IF USED AS PART OF A REVIEW PROCEDURE FOR PUBLICATION, IT SHOULD NOT REPLACE THE EXPERTISE OF AN EXPERIENCED CRYSTALLOGRAPHIC REFEREE.

No syntax errors found.   CIF dictionary   Interpreting this report

Datablock: I

```
Bond precision:  Si- O = 0.0050 A        Wavelength=0.48300

Cell:               a=4.194(2)      b=4.525(1)      c=6.1910(1)
                    alpha=90        beta=90         gamma=90
Temperature:        293 K

                Calculated                  Reported
Volume          117.49(6)                   117.49(6)
Space group     P b n m                     P b n m
Hall group      -P 2c 2ab                   -P -2xab;-2
Moiety formula  Fe0.36 Mg3.64 O12 Si4       Mg0.909 Fe0.091 Si1 O3
Sum formula     Fe0.36 Mg3.64 O12 Si4       Mg0.909 Fe0.091 Si1 O3
Mr              413.08                      103.30
Dx,g cm-3       5.838                       5.838
Z               1                           4
Mu (mm-1)       1.015                       0.984
F000            205.1                       205.0
F000’           205.39
h,k,lmax        5,5,7                       4,5,7
Nref            122                         56
Tmin,Tmax                                   0.664,1.000
Tmin’

Correction method= # Reported T Limits: Tmin=0.664 Tmax=1.000
AbsCorr = MULTI-SCAN

Data completeness= 0.459          Theta(max)= 17.070

R(reflections)= 0.0412( 53)       wR2(reflections)= 0.1146( 56)

S = 3.550                  Npar= 13
```

The following ALERTS were generated. Each ALERT has the format
test-name_ALERT_alert-type_alert-level.
Click on the hyperlinks for more details of the test.

Alert level A

```
ATOM007_ALERT_1_A  _atom_site_aniso_label is missing
            Unique label identifying the atom site.
```

Author Response: Due to incompleteness of the dataset thermal parameters were refined in isotropic approximation.

```
PLAT027_ALERT_3_A _diffrn_reflns_theta_full value (too) Low ......      12.34 Degree
```

Author Response: The dataset was incomplete since the data were collected in a diamond anvil cell metallic body of which shadows more than 60% of the reflections

```
PLAT029_ALERT_3_A _diffrn_measured_fraction_theta_full value Low .      0.650 Why?
```

Author Response: The dataset was incomplete since the data were collected in a diamond anvil cell metallic body of which shadows more than 60% of the reflections.

Alert level C

```
GOODF01_ALERT_2_C  The least squares goodness of fit parameter lies
            outside the range 0.80 <> 2.00
            Goodness of fit given =      3.550
PLAT041_ALERT_1_C Calc. and Reported SumFormula    Strings  Differ     Please Check
PLAT077_ALERT_4_C Unitcell Contains Non-integer Number of Atoms ..     Please Check
PLAT088_ALERT_3_C Poor Data / Parameter Ratio ....................       9.38 Note
PLAT127_ALERT_1_C Implicit Hall Symbol  Inconsistent with Explicit  -P -2xa  b;-2yab
```

Alert level G

```
ABSMU01_ALERT_1_G  Calculation of _exptl_absorpt_correction_mu
                not performed for this radiation type.
PLAT004_ALERT_5_G Polymeric Structure Found with Maximum Dimension          3 Info
PLAT005_ALERT_5_G No Embedded Refinement Details Found  in the CIF     Please Do !
PLAT042_ALERT_1_G Calc. and Reported MoietyFormula Strings  Differ     Please Check
PLAT045_ALERT_1_G Calculated and Reported Z Differ by a Factor ...       0.25 Check
PLAT092_ALERT_4_G Check: Wavelength Given is not Cu,Ga,Mo,Ag,In Ka    0.48300 Ang.
PLAT128_ALERT_4_G Alternate Setting for Input Space Group  Pbnm          Pnma Note
PLAT199_ALERT_1_G Reported _cell_measurement_temperature ..... (K)        293 Check
PLAT200_ALERT_1_G Reported   _diffrn_ambient_temperature ..... (K)        293 Check
PLAT301_ALERT_3_G Main Residue  Disorder ..............(Resd  1  )        30% Note
PLAT432_ALERT_2_G Short Inter X...Y Contact  Si1      ..O2               2.99 Ang.
                                                -x,-y,1-z  =      5_556 Check
PLAT432_ALERT_2_G Short Inter X...Y Contact  Si1      ..O2               2.99 Ang.
                                                  1+x,y,z  =      1_655 Check
PLAT432_ALERT_2_G Short Inter X...Y Contact  Si1      ..O1               3.07 Ang.
                                               1-x,-y,1-z  =      5_656 Check
PLAT432_ALERT_2_G Short Inter X...Y Contact  Si1      ..Si1              3.08 Ang.
                                          1/2-x,-1/2+y,z  =      8_545 Check
```

```
PLAT432_ALERT_2_G Short Inter X...Y Contact  Si1      ..Si1              3.08 Ang.
                                         3/2-x,-1/2+y,z  =      8_645 Check
PLAT432_ALERT_2_G Short Inter X...Y Contact  Si1      ..Si1              3.10 Ang.
                                          1-x,-y,-1/2+z  =      2_654 Check
PLAT432_ALERT_2_G Short Inter X...Y Contact  Si1      ..Si1              3.10 Ang.
                                           1-x,-y,1/2+z  =      2_655 Check
PLAT432_ALERT_2_G Short Inter X...Y Contact  Si1      ..O2               3.17 Ang.
                                          1-x,-y,-1/2+z  =      2_654 Check
PLAT432_ALERT_2_G Short Inter X...Y Contact  Si1      ..O2               3.17 Ang.
                                               x,y,3/2-z  =      6_556 Check
PLAT432_ALERT_2_G Short Inter X...Y Contact  Si1      ..O2               3.17 Ang.
                                     1/2-x,-1/2+y,3/2-z  =      3_546 Check
PLAT432_ALERT_2_G Short Inter X...Y Contact  Si1      ..O2               3.23 Ang.
                                       1/2+x,-1/2-y,1-z  =      4_546 Check
PLAT883_ALERT_1_G No Info/Value for _atom_sites_solution_primary .     Please Do !
PLAT985_ALERT_1_G The Fe-f"=    0.4167 Deviates from the B&C-Value     0.4134 Check
```

```
   3 ALERT level A = Most likely a serious problem - resolve or explain
   0 ALERT level B = A potentially serious problem, consider carefully
   5 ALERT level C = Check. Ensure it is not caused by an omission or oversight
  23 ALERT level G = General information/check it is not something unexpected

  10 ALERT type 1 CIF construction/syntax error, inconsistent or missing data
  12 ALERT type 2 Indicator that the structure model may be wrong or deficient
   4 ALERT type 3 Indicator that the structure quality may be low
   3 ALERT type 4 Improvement, methodology, query or suggestion
   2 ALERT type 5 Informative message, check
```

It is advisable to attempt to resolve as many as possible of the alerts in all categories. Often the minor alerts point to easily fixed oversights, errors and omissions in your CIF or refinement strategy, so attention to these fine details can be worthwhile. In order to resolve some of the more serious problems it may be necessary to carry out additional measurements or structure refinements. However, the purpose of your study may justify the reported deviations and the more serious of these should normally be commented upon in the discussion or experimental section of a paper or in the "special_details" fields of the CIF. checkCIF was carefully designed to identify outliers and unusual parameters, but every test has its limitations and alerts that are not important in a particular case may appear. Conversely, the absence of alerts does not guarantee there are no aspects of the results needing attention. It is up to the individual to critically assess their own results and, if necessary, seek expert advice.

Publication of your CIF in IUCr journals

A basic structural check has been run on your CIF. These basic checks will be run on all CIFs submitted for publication in IUCr journals (*Acta Crystallographica*, *Journal of Applied Crystallography*, *Journal of Synchrotron Radiation*); however, if you intend to submit to *Acta Crystallographica Section C* or *E* or *IUCrData*, you should make sure that full publication checks are run on the final version of your CIF prior to submission.

Publication of your CIF in other journals

Please refer to the *Notes for Authors* of the relevant journal for any special instructions relating to CIF submission.

PLATON version of 05/12/2020; check.def file version of 05/12/2020

Datablock I - ellipsoid plot

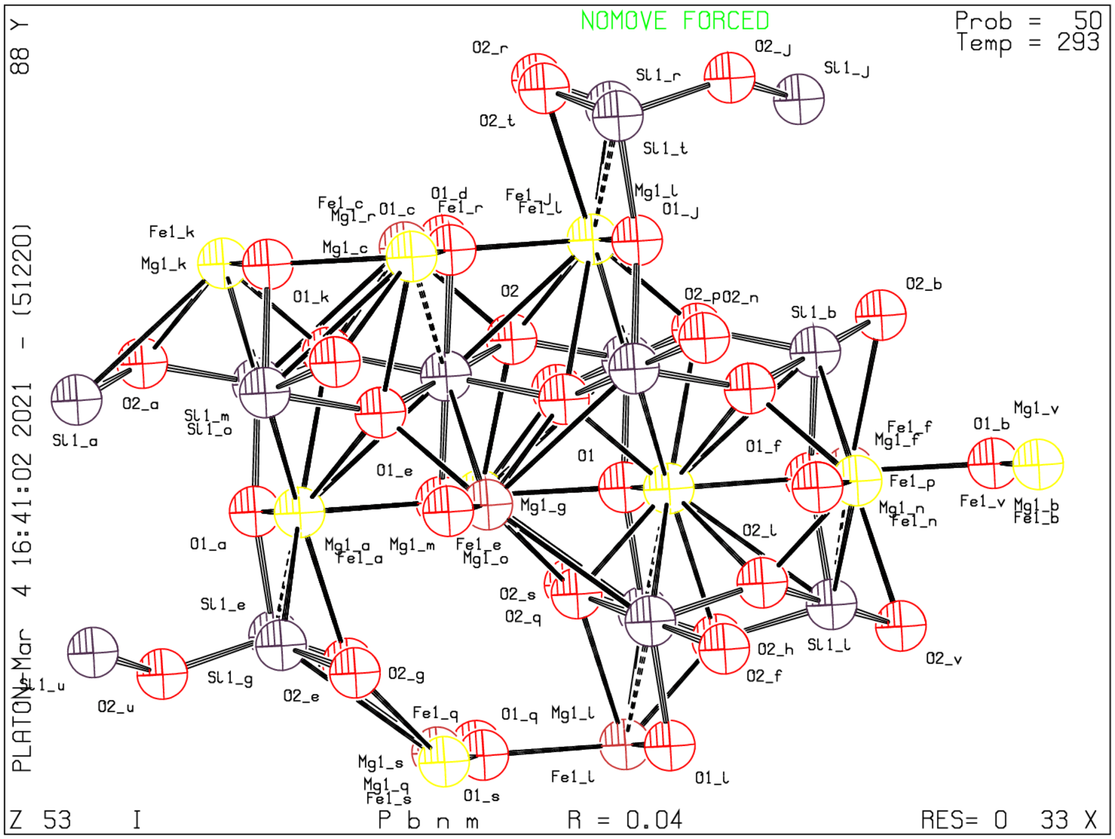

Supplement: Supplementary file 4 [file checkcif_Table3_bridgmanite_103GPa.pdf › 04_bridgmanite_155GPa/checkcif_Table3_bridgmanite_103GPa.pdf]
